# Oligopeptide-binding protein A is a potential target for combating *Salmonella* infection

**DOI:** 10.3389/fimmu.2026.1943609

**Published:** 2026-10-09

**Authors:** Ken Yoshii, Koichiro Suzuki, Jun Adachi, Zhenyu Piao, Eri Node, Zilai Liu, Takahiro Nagatake, Koji Hosomi, Jun Kunisawa

**Affiliations:** 1 Laboratory of Vaccine Materials and Laboratory of Gut Environmental System, Microbial Research Center for Health and Medicine, National Institutes of Biomedical Innovation, Health and Nutrition (NIBN), Osaka, Japan; 2 Biomanufacturing Process Research Center, National Institute of Advanced and Industrial Science and Technology (AIST), Ibaraki, Japan; 3 The Research Foundation for Microbial Diseases of The University of Osaka, Osaka, Japan; 4 BIKEN-RIMD NGS Laboratory, Research Institute for Microbial Diseases, The University of Osaka, Osaka, Japan; 5 Laboratory of Proteomics for Drug Discovery, National Institutes of Biomedical Innovation, Health and Nutrition (NIBN), Osaka, Japan; 6 Graduate School of Pharmaceutical Sciences, The University of Osaka, Osaka, Japan; 7 Laboratory of Functional Anatomy, Department of Life Sciences, School of Agriculture, Meiji University, Kawasaki, Kanagawa, Japan; 8 Graduate School of Veterinary Science, Osaka Metropolitan University, Osaka, Japan; 9 Graduate School of Medicine, The University of Osaka, Osaka, Japan; 10 Graduate School of Dentistry, The University of Osaka, Osaka, Japan; 11 Graduate School of Science, The University of Osaka, Osaka, Japan; 12 International Vaccine Design Center, The Institute of Medical Science, The University of Tokyo, Tokyo, Japan; 13 Department of Microbiology and Immunology, Kobe University Graduate School of Medicine, Kobe, Hyogo, Japan; 14 Graduate School of Biomedical and Health Sciences, Hiroshima University, Hiroshima, Japan; 15 Research Organization for Nano and Life Innovation, Waseda University, Tokyo, Japan

**Keywords:** energy metabolism, monoclonal antibody, mucosal vaccine, neutrophil, OppA, salmonellosis

## Abstract

**Introduction:**

*Salmonella* infection remains a pervasive diarrheal illness, particularly affecting populations in developing countries. The lack of an effective vaccine against non-typhoidal *Salmonella* is attributable to antigenic diversity among serotypes, incomplete understanding of protective immune mechanisms, and the difficulty in identifying conserved antigens capable of eliciting broad cross-protective immunity.

**Methods:**

To identify conserved *Salmonella* antigens, we established a *Salmonella*-specific monoclonal antibody library using heat-killed bacteria. Candidate antigens recognized by monoclonal antibodies were characterized, and their functional relevance was evaluated through analyses of bacterial growth, respiratory activity, and protective effects in a murine *Salmonella* infection model.

**Results:**

We identified L2-1H11, a monoclonal antibody that recognizes OppA, a conserved antigen present across multiple *Salmonella* serotypes. L2-1H11 was associated with suppression of bacterial growth and respiratory activity and attenuated enteritis pathology in *Salmonella*-infected mice.

**Conclusion:**

These findings identify OppA as a conserved *Salmonella* antigen and suggest that it may represent a promising target for the development of subcutaneous and oral vaccines against *Salmonella*.

## Introduction

*Salmonella* are gram-negative motile rod-shaped bacteria that infect approximately 93 million individuals globally each year (1). Certain *Salmonella* serotypes cause salmonellosis, a debilitating gastrointestinal condition that manifests primarily as enteritis and diarrhea and causes over 150,000 fatalities annually (2). In severe instances, *Salmonella* can disseminate to vital organs such as the liver and spleen, culminating in fatal septicemia. Some *Salmonella* serotypes are also zoonotic and can be transmitted from animals to humans. Furthermore, injudicious use of antibiotics is threatening the development of antimicrobial-resistant strains. Thus, concerted efforts are warranted to confront these multifaceted challenges and safeguard global health.

The *Salmonella* genus includes *S. enterica* and *S. bongori* and approximately 2650 serotypes, distinguished by variations in their O and H antigens (3). *Salmonella enterica* subspecies *enterica* comprises both typhoidal and non-typhoidal serotypes. Among them, non-typhoidal *Salmonella* serotypes infect a broad range of animals and humans and are major causes of zoonotic gastroenteritis worldwide. Infection by *Salmonella* begins with its adhesion to intestinal epithelial cells, followed by the secretion of effector molecules into the epithelial cells via the type III secretion system and bacterial invasion. Then, production of inflammatory cytokines such as interleukin (IL)-6, IL-1β, and tumor necrosis factor α increases in intestinal epithelial cells, along with the induction of cell death (4). This induction of cell death disrupts the epithelial barrier, leading to infiltration of neutrophils and macrophages into the lamina propria, ultimately contributing to the pathogenesis of salmonellosis (5).

The development of vaccines and therapeutic agents against *Salmonella* infection that target adhesins, flagellin, lipopolysaccharides (LPS), and other molecules involved in adhesion of *Salmonella* to intestinal epithelial cells has been proposed. For example, the outer membrane adhesin T2544 has been proposed as a subunit vaccine candidate capable of inducing systemic and mucosal immune responses (6). Flagellin, beyond its role in motility, is a Toll-like receptor 5 agonist and has been exploited as both an antigen and adjuvant (7). In addition, O-antigen–based conjugate and generalized modules for membrane antigen platforms have been shown to elicit bactericidal antibodies (8), supporting adhesion-associated molecules as promising targets for next-generation interventions. Administering a recombinant oral vaccine expressing FliC, a type of flagellin from *Salmonella enterica* subsp. *enterica* serotype Enteritidis, on the surface of *Lactobacilli* or *Escherichia coli* to mice has induced *S.* Enteritidis–specific IgG and IgA antibody production (9). Administering mAb-449, a monoclonal IgG antibody that recognizes *S*. Typhimurium LPS, via the tail vein in mice has prevented oral infection by *S*. Typhimurium and increased survival rates (10). However, these interventions are effective only against specific *Salmonella* serotypes. Thus, finding broad-spectrum *Salmonella* vaccine antigens for humans remains an ongoing challenge.

In the present study, we used immunization with heat-killed *Salmonella* bacteria to establish an antibody library without bias towards specific target molecules. Using this library, we conducted comprehensive screening based on reactivity against various *Salmonella* serotypes and key processes in infection (i.e., adhesion, invasion, and intestinal epithelial cell death). We identified an antibody with broad cross-reactivity to multiple *Salmonella* serotypes and the ability to prevent infection. Further characterization revealed the mechanisms of the activity of the antibody and the possibility of developing vaccines that target the protein recognized by this antibody.

## Methods details

### Study approval

Female BALB/c mice (age, 7 weeks) were purchased from CLEA, Inc. and kept for at least 1 week before experiments. Mice were housed with a 12-h light/12-h dark cycle and were allowed free access to food and water. Animal experiments were approved by the Animal Care and Use Committee of the National Institutes of Biomedical Innovation, Health and Nutrition (Osaka, Japan; approval no. DSR04-37R7 and DSR04-38R7), and were conducted in accordance with its guidelines and ARRIVE guidelines.

### Development of *Salmonella*-specific hybridomas

Development of *Salmonella*-specific hybridomas were performed as previously described (11) with modification. Briefly, Heat-killed cells of *S.* Enteritidis (RIMD1933006) or *S.* Typhimurium (RIMD1985055) were suspended in phosphate-buffered saline (PBS) at a concentration of 1.0 × 10^8^ colony-forming units [CFU]/mouse, mixed with Sigma Adjuvant System (Sigma-Aldrich), and then subcutaneously injected once a week for 2 consecutive weeks into BALB/c mice (female, 8 weeks old) purchased from Japan CLEA. One week after the second injection, the spleen and inguinal and popliteal lymph nodes were harvested, crushed, passed through a 100-μm cell strainer (Corning), and subjected to red blood cell lysis with lysis buffer. Hybridomas were then established by fusion of the immune cells with P3U1 myeloma cells using polyethylene glycol 1500 (Roche). The hybridomas were suspended in Dulbecco’s Modified Eagle medium (DMEM; high glucose; Nacalai Tesque) (composition: 10% fetal bovine serum [Gibco], 1% penicillin-streptomycin [Nacalai Tesque], 1% 100 mM sodium pyruvate solution and 1% MEM non-essential amino acid solution [Nacalai Tesque], 0.1% 2-mercaptoethanol [Gibco], 10 ng/mL recombinant murine IL-6 [PeproTech]) containing hypoxanthine-aminopterin-thymidine (HAT) supplement (MP Biomedicals), seeded at 100 µL/well in flat-bottom 96-well plates for cell suspensions (Sumitomo Bakelite), and cultured at 37 °C in a 10% CO_2_ incubator. At 10–14 days of culture, the supernatant was subjected to ELISA to determine if the antibodies in the supernatant were specific to *Salmonella*.

### Screening for *Salmonella*-specific clones

Screening of *Salmonella*-specific monoclonal antibodies were performed as previously described (12) with modification. In brief, heat-killed cells of *S.* Enteritidis and *S*. Typhimurium suspended in PBS were seeded at 0.1 mg/mL in 96-well immunoplates (Thermo Fisher Scientific) and incubated overnight at 4 °C. Bovine serum albumin (1%; Nacalai Tesque) dissolved in PBS was seeded at 170 µL/well in 96-well immunoplates and incubated at room temperature (25 °C) for 2 h and the wells were washed three times with 0.05% Tween-20–PBS. The culture supernatant of the hybridomas was added at 50 μL/well, incubated at 25 °C for 2 h and the wells were washed three times with 0.05% Tween-20–PBS. Horseradish peroxidase–labeled goat anti-mouse IgG antibody (Southern Biotech) diluted 1:4000 in 1% bovine serum albumin–0.05% Tween-20–PBS was added at 100 µL/well and incubated at 25 °C for 1 h. *Salmonella*-specific antibodies were detected by adding 3,3’,5,5’-tetramethylbenzidine peroxidase substrate (SeraCare Life Sciences) at 100 µl/well and incubating for 2 min. Then, 0.5 M HCl was added at 50 µl/well, and the absorbance at 450 nm (OD_450_) was measured in an iMark Microplate Reader (Bio-Rad Laboratories). Based on the absorbance, hybridomas with excellent *Salmonella* specificity were selected, suspended in Nacalai Tesque’s DMEM high-glucose medium containing HAT supplement, subjected to limiting dilution, seeded in flat-bottom 96-well plates for suspension cells (Sumitomo Bakelite), and single-cell cloning was performed. After 10 days of culture, the supernatant was used to screen for monoclonal antibodies with excellent *Salmonella* specificity by ELISA as described above.

### Purification of monoclonal antibody

Hybridomas were suspended in Hyclone ADCF-MAb medium (Cytiva) at 1.0 × 10^6^ cells/mL, seeded into 175-cm^2^ flasks (Greiner Bio-One), and cultured at 37 °C in a 10% CO_2_ incubator. After 2 days, the culture supernatant was collected and filtered using a 0.22-µm filter system (Corning). Protein G Sepharose (Nacalai Tesque) was packed into Econo-Columns (Bio-Rad Laboratories), and then equilibrated with binding buffer (20 mM sodium phosphate, pH 7.0) at a flow rate of 1.0 mL/min. The filtered supernatant was added to the column. The column was washed with binding buffer, and the antibody was eluted with elution buffer (0.1 M glycine-HCl, pH 3.0). The eluate (450 µL) was collected in an Eppendorf tube containing 50 µL of neutralization buffer (1.0 M Tris-HCl, pH 9.0). The absorbance (A_280_) of the eluted fractions was measured using a microspectrophotometer (Scrum Inc.). The collected fractions were concentrated by centrifugation at 4 °C, 5000 × *g* using an Amicon Ultra-5 (30K; Merck Millipore) with the addition of PBS at 5 mL; the procedure was repeated three times, and the absorbance (A_280_) of the concentrated solution was measured using the microspectrophotometer. The purified L2-1H11 monoclonal antibody was identified as an IgG1 isotype using a Rapid Mouse Immunoglobulin Isotyping Kit (Antagen) according to the manufacturer’s instructions. An isotype-matched mouse IgG1 antibody was used as the negative control in all antibody-based experiments.

### ELISA

L2-1H11 was used to test specificity and cross-reactivity by ELISA as described above. Briefly, 96-well immunoplates (Thermo Fisher Scientific) were coated with lyophilized heat-killed bacteria (1 mg/mL, 100 µL/well) diluted with PBS or *S.* Typhimurium- or *E. coli*-derived recombinant OppA (5 µg/mL, 100 µL/well) diluted with PBS at 4 °C overnight. The plates were blocked with 1% (w/v) bovine serum albumin in PBS for 2 h at room temperature to prevent non-specific binding and washed with PBS containing 0.05% Tween 20 (PBS-T; Nacalai Tesque). Purified L2-1H11 monoclonal antibody (1 µg/mL, 100 µL/well) was added to the wells, and the plates were incubated for 2 h at room temperature. The plates were washed with PBS-T and incubated with horseradish peroxidase–conjugated goat anti-mouse IgG for 1 h at room temperature and washed with PBS-T. Tetramethylbenzidine peroxidase substrate was added to the wells, and the plates were incubated for 2 min. Absorbance was measured at 450 nm using an iMark microplate reader (Bio-Rad Laboratories). Lyophilized heat-killed cells as antigens for ELISA were as follows: *S.* Enteritidis (RIMD1933006), *S.* Typhimurium (RIMD1985055), *S.* Infantis (RIMD1905012), *S.* Thompson (RIMD1982025), *S*. Schwarzengrund (RIMD3178001) provided by the RIMD-PMRU through the Pathogenic Bacteria program in the National BioResource Project of MEXT. *Campylobacter jejuni* strain 81–176 was purchased from a commercial supplier and used as the reference pathogenic strain in this study. *E. coli* DH5α (Competent Quick DH5α; Toyobo, Osaka, Japan) was commercially obtained and used. *E. coli* O111 (NBRC IID561) was obtained from the Pathogenic Microbes Repository Unit (Tokyo, Japan). *Bifidobacterium longum* subsp. *longum* (JCM1217), *Bifidobacterium pseudocatenulatum* (JCM1200), *Phocaeicola vulgatus* (JCM5826), *Faecalibacterium duncaniae* (JCM31915), *Segatella copri* (JCM13464), *Lactobacillus gasseri* (JCM1131), *Pseudomonas aeruginosa* (JCM5962T), *Staphylococcus aureus* (JCM2151), *Serratia liquefaciens* (JCM1245T), *Proteus mirabilis* (JCM1235T), *Citrobacter freundii* (JCM1657T), *Escherichia hermannii* (JCM1473), *Klebsiella pneumoniae* (JCM1662T), *Klebsiella aerogenes* (JCM1235T), *Enterobacter cloacae* (JCM1232T), *Enterococcus faecalis* (JCM5803T) provided by the RIKEN BRC through the National BioResource Project of MEXT/AMED.

### Immunoprecipitation and proteome analysis

*S.* Typhimurium was suspended in B-PER Complete Bacterial Extraction Reagent (Thermo Fisher Scientific) supplemented with 0.5 M EDTA (Nacalai Tesque) to a final concentration of 1 mM, incubated at 25 °C for 15 min, and centrifuged at 6000 × *g* for 20 min at 25 °C. The supernatant was collected and mixed with L2-1H11 antibody and Protein G Sepharose (Nacalai Tesque), incubated at 4 °C for 1 h, and centrifuged at 12,000 × *g* for 20 s at 4 °C. The pellet was subjected to SDS-PAGE using a 4%–12% Bis-Tris gel and MES buffer with a NuPAGE electrophoresis system (Life Technologies). SeeBlue Plus2 Pre-Stained Standard (Thermo Fisher Scientific) was used as the molecular weight marker. The gel was silver stained using a Pierce Silver Stain Kit (Thermo Fisher Scientific). The bands of interest were excised and digested with trypsin to generate peptide fragments, essentially as described (13). Briefly, the gel pieces were destained and washed, and after dithiothreitol reduction and iodoacetamide alkylation, the proteins were digested with porcine trypsin (modified sequencing grade; Promega, Madison, Wisconsin, United States) overnight at 37 °C. The resulting tryptic peptides were extracted from the gel pieces consecutively with 30% acetonitrile containing 0.3% trifluoroacetic acid, then 100% acetonitrile. The extracts were evaporated in a vacuum centrifuge to remove organic solvent, then desalted and concentrated on reversed-phase C18 StageTips (14), followed by proteome analysis using a mass spectrometer.

Liquid chromatography–tandem mass spectrometry was performed with an UltiMate 3000 Nano LC system (Thermo Fisher Scientific) and an HTC-PAL autosampler (CTC Analytics) coupled to a Q Exactive mass spectrometer (Thermo Fisher Scientific). Gradients (45 min) from 5% to 30% Buffer B (0.1% formic acid and 90% acetonitrile) were used. The Q Exactive instrument was operated as previously described (15). Raw mass spectrometry data were processed with the MaxQuant (1.6.14.0) using the Andromeda search engine and a UniProt *S.* Typhimurium (Uniprot Proteome ID: UP000001014). The following parameter settings were used in the search: fully tryptic peptides with a minimum of 7 amino acids, maximum 2 missed tryptic cleavage sites, tolerances of 10 ppm for precursor ions and 0.01 Da for-fragment ions, all b/y/a ions considered in the search, and dynamic modifications allowed on the N-terminus (42.0106 Da/acetyl) and methionine (15.9949 Da/oxidation). For peptide-spectrum match validation, a forward/reverse concatenated database was used. Only first-rank peptides were counted, and only for top-scoring proteins. The probability thresholds for all peptide/protein level False Discovery Rates were set at confidence thresholds of 0.05 (relaxed) or 0.01 (stringent).

### Production of recombinant *S. typhimurium*- or *E. coli*-derived OppA protein

Recombinant protein was prepared as previously described (11, 16). Briefly, *S.* Typhimurium–derived OppA fragment was obtained by DNA amplification through PCR with a forward primer 5′-GGGCATATGGGCAGACGTGCCCGCCGGCGTTCAG-3′ (NdeI site is underlined) and a reverse primer 5′-GGGGATCCTTAATGTTTGATAATATATAAGTTTTTC-3′ (BamHI site is underlined). *E. coli–*derived OppA fragment was obtained by DNA amplification through PCR with a forward primer 5′-GGGCATATGATGACCAACATCACCAAGAGAAG-3′ (NdeI site is underlined) and a reverse primer 5′-GGGGGATCCTTAGTGCTTCACAA-3′ (BamHI site is underlined). The pET16b (Novagen) vector and the OppA fragment were each digested with NdeI (New England Biolabs; NEB) and BamHI (NEB) and ligated using Ligation High Ver.2 (Toyobo) to create the pET16b-OppA plasmid. This plasmid was transformed into *E*. *coli* strain BL21 (DE3), and protein expression was induced by adding isopropyl-β-d-thiogalactopyranoside (Nacalai Tesque) at 0.5 mM to the bacterial cell culture, shaken at 150 rpm/min and incubated at 37 °C for 3 h. The culture medium was centrifuged at 4 °C, 3,020 × *g* for 10 min to collect the pellet. The pellets were suspended in Buffer A (10 mM Tris-HCl [pH 8.0], 400 mM NaCl, 5 mM MgCl_2_, 0.1 mM phenylmethylsulfonyl fluoride, 1 mM 2-mercaptoethanol, and 10% glycerol) and sonicated three times (1 min each). The suspension was centrifuged at 4 °C, 17,800 × *g* for 15 min, and the supernatant was filtered through a 0.45-μm filter (Merck Millipore). Protein was purified on a HisTrap HP column (Cytiva) mounted on an NGC chromatography system (Bio-Rad Laboratories). OppA was eluted in Buffer A containing 100–500 mM imidazole, and the buffer was exchanged to PBS using a PD-10 column (Cytiva). The concentration of purified protein was measured using a BCA protein assay kit (Thermo Fisher Scientific), and its purity was confirmed by performing SDS-PAGE using a 4%–12% Bis-Tris gel and MES buffer in a NuPAGE electrophoresis system (Life Technologies), followed by staining with Coomassie Brilliant Blue (EzStainAQua, ATTO). SeeBlue Plus2 Pre-Stained Standard was used as the molecular weight marker.

### Western blotting

*S.* Typhimurium-derived recombinant OppA protein (2 µg per lane) and immunoprecipitated samples were subjected to SDS-PAGE (as above). The gel was transferred to a polyvinylidene fluoride membrane (EMD Millipore Corporation). The membrane was immersed in 4% skimmed milk dissolved in 0.05% Tween-20–PBS overnight at 4 °C. L2-1H11 monoclonal antibody (40 ng/mL) diluted in Can Get Signal Solution 1 (Toyobo) was used as the primary antibody and then incubated with the membrane for 1 h at 25 °C. The membrane was washed three times with 0.05% Tween-20–PBS for 5 min each time. Horseradish peroxidase–labeled anti-mouse IgG diluted 1: 5000 in Can Get Signal Solution 2 (Toyobo) was used as the secondary antibody, and then incubated with the membrane for 1 h at 25 °C. The membrane was washed three times with 0.05% Tween-20–PBS for 5 min each time. Chemiluminescence signal generated by horseradish peroxidase was detected using an ImageQuant LAS 4000 biomolecular imager (Fujifilm) to visualize the band of the target protein.

### Measurement of ATP concentration

*S.* Typhimurium was cultured in LB broth at 37 °C and 200 rpm/min until 1.0 × 10^8^ CFU/mL. Culture medium was centrifuged 10,000 × *g* for 5 min at 25 °C. The pellet was diluted in PBS and incubated with 100 µg of L2-1H11 antibody at 37 °C for 1 h. After the incubation and collecting samples over time, the ATP concentration within the *Salmonella* cells was measured using the BacTiter-Glo Microbial Cell Viability Assay (Promega) according to the manufacturer’s instruction.

### *In vitro* Salmonella infection model

Human intestinal epithelial cell line HT29 was suspended in DMEM high-glucose medium containing 10% fetal bovine serum and 1% non-essential amino acids (Nacalai Tesque) to 1.0 × 10^5^ cells/mL. The cells were seeded at 1.0 × 10^5^ cells/well in a 12-well plate (Corning) and cultured overnight. *S.* Typhimurium, pre-bound with L2-1H11 antibody or Ultra-LEAF™ Purified Mouse IgG1, κ Isotype Ctrl Antibody (400166, BioLegend) as described above, was then used to infect the cells at a multiplicity of infection of 10.

### Lactate dehydrogenase assay

After 12 h of infecting HT29 cells with *S.* Typhimurium, the supernatant was collected, and the amount of lactate dehydrogenase released during cell death was measured using a CytoTox 96 Non-Radioactive Cytotoxicity Assay kit (Promega) according to the manufacturer’s protocol to calculate the percentage of cell death.

### Cell association assay

Cells were infected with *S.* Typhimurium for 30 min at 37 °C to allow bacterial attachment and initial entry. Cells were washed twice with PBS to remove non-adherent bacteria. Cells were detached using trypsin-EDTA solution (2.5 g/L trypsin and 1 mM EDTA; Nacalai Tesque), incubated at 37 °C for 10 min, collected, and plated onto *Salmonella-Shigella* agar (Becton Dickinson). Plates were incubated overnight at 37 °C, and colonies were counted. This assay quantifies total cell-associated bacteria.

### Invasion assay

To quantify intracellular bacteria, cells were infected with *S.* Typhimurium for 1 h at 37 °C. The medium was replaced with DMEM high glucose supplemented with 100 µg/mL gentamicin and 10% fetal bovine serum for 1 h to kill extracellular bacteria. Cells were washed twice with PBS, detached using trypsin-EDTA and lysed in PBS containing 1% Triton X-100 (Nacalai Tesque) at 25 °C for 10 min. The lysates were plated onto *Salmonella-Shigella* agar and incubated overnight at 37 °C. Colonies were counted to determine the number of gentamicin-protected intracellular bacteria.

### Preparation of bone marrow–derived macrophages and phagocytosis assay

Bone marrow–derived macrophages were prepared as follows. Briefly, BALB/c mice (4 weeks old) were killed, and bone marrow cells were extracted from the femurs and tibias. These cells were treated with red blood cell lysis buffer (0.16 mol/L NH_4_Cl and 0.17 mol/L Tris) for 1 min at room temperature. The cells were cultured in 6-cm dishes (1×10^5^ cells/mL, 5 mL/dish; RepCell dishes; CellSeed) with DMEM (high glucose) supplemented with macrophage colony–stimulating factor (50 ng/mL; PeproTech), 10% (vol/vol) fetal bovine serum (Corning, Corning, NY, USA), and 1% (vol/vol) penicillin–streptomycin (Nacalai Tesque) at 37 °C in 5% CO_2_ (day 0). Culture medium was replaced on days 3 and 5. On day 6, cells were seeded into 24-well plates (Corning) at 1×10^5^ cells/well, centrifuged (200 × *g*, 10 min, room temperature) for adhesion, and incubated in DMEM (high glucose) containing 10% (vol/vol) fetal bovine serum and 1% (vol/vol) penicillin–streptomycin at 37 °C under 5% CO_2_ overnight. *S.* Typhimurium were grown in LB broth overnight at 37 °C with shaking at 200 rpm. They were incubated with 5.0 mg/mL FITC (Sigma Aldrich) for 1 h at 37 °C in a water bath and protected from light and were then washed twice with PBS. Cells were centrifuged (200 × *g*, 10 min, room temperature) for adhesion and incubated with the FITC-stained *S.* Typhimurium at cell/bacteria ratio of 1:10 for 30 min. Non-phagocytosed bacteria were removed by washing three times with PBS, and the cells were harvested by treating with 0.5 g/L trypsin–0.53 mmol/L EDTA (Nacalai Tesque) for 5 to 10 min at 37 °C in 5% CO_2_. Phagocytosis activity was assessed by calculating the mean fluorescence intensity from flow cytometric analysis.

### *In vivo* Salmonella infection model

Female BALB/c mice (8 weeks old) were orally administered streptomycin (Nacalai Tesque) dissolved in ultra-pure water to achieve a concentration of 20 mg/mouse to inhibit microbiota-associated intestinal colonization resistance against *S.* Typhimurium. At 24 h after administration of streptomycin, the mice were fasted (>4h~) and orally administered 100 µl of 10% sodium bicarbonate (Nacalai Tesque) dissolved in ultrapure water to neutralize gastric acid. After these treatments, mice were orally infected with *S.* Typhimurium at 1.0 × 10^6^ CFU/mouse, which had been pre-treated with 100 µg of L2-1H11 antibody, Ultra-LEAF™ Purified Mouse IgG1, κ Isotype Ctrl Antibody, or anti-*S.* Typhimurium LPS antibody (ab8274, Abcam) for 1 h at 37 °C or not, suspended in 500 μL of PBS.

Purified L2-1H11 antibody or Ultra-LEAF™ Purified Mouse IgG1, κ Isotype Ctrl Antibody (each at 100 µg/mouse) was administered intravenously via the tail vein to female BALB/c mice (8 weeks old). On the following day, *S.* Typhimurium (1.0 × 10^6^ CFU/mouse) was injected intravenously via the tail vein. Survival and body weight were monitored daily for one week after infection.

### *Salmonella*-specific gene expression analysis by quantitative PCR

*S.* Typhimurium was cultured aerobically at 37 °C overnight in LB broth and collected by centrifugation at 10,000 × *g* for 5 min at 25 °C. The bacterial cells (10^8^ CFU) were incubated with 1 µg of L2-1H11 monoclonal antibody dissolved in PBS for 1 h at 37 °C. The cells were washed three times with PBS, and then RNA from *S.* Typhimurium was extracted by using PureLink RNA Mini kit (Invitrogen) according to the manufacturer’s instructions. After extraction, extracted samples were treated with DNase I solution (Invitrogen) to remove genome DNA and then reverse-transcribed into cDNA using the VIRO cDNA Synthesis Kit (Invitrogen).

Quantitative PCR analysis was performed by using a CFX Opus Real-Time PCR System (Bio-Rad Laboratories) with TB Green Premix Ex Taq II (Takara Bio). Primer sequences were as follows: *fliC* forward primer 5′-ATAGCCATCTTTACCAGTTCCCCC-3′, *fliC* reverse primer 5′-GCTGCAACTGTTACAGGATATGCC-3′, *fljB* forward primer 5′-ACGAATGGTACGGCTTCTGTAACC-3′, *fljB* reverse primer 5′- TACCGTCGATAGTAACGACTTCGG-3′, *fimA* forward primer 5′-TGCCTTTCTCCATCGTCC-3′, *fimA* reverse primer 5′-TGCGGTAGTGCTATTGTCC-3′, *fimH* forward primer 5′-ATCCCTCGCCAGACAATG-3′, *fimH* reverse primer 5′-TCGCCGAAATCAAACTCC -3′, *invH* forward primer 5′-AGCAACTGGCCAACGCAAAT -3′, *invH* reverse primer 5′-TGCAGTCTTTCATGGGCAGCAA-3′, *S.* Typhimurium 16s forward primer 5′-ATGGCTCAGATTGAACGC-3′, *S.* Typhimurium 16s reverse primer 5′-GGCAGTTTCCCAGACATTAC-3′.

### Pulse chase assay

*S.* Typhimurium was cultured aerobically at 37 °C overnight in LB broth and collected by centrifugation at 10,000 × *g* for 5 min at 25 °C. The bacterial cells (10^8^ CFU) were incubated with 1 µg of L2-1H11 monoclonal antibody or Ultra-LEAF™ Purified Mouse IgG1, κ Isotype Ctrl Antibody dissolved in PBS for 1 h at 37 °C and then washed three times with PBS. Protein synthesis of *S.* Typhimurium with or without L2-1H11 antibody was investigated by using the Click-iT HPG Alexa Fluor protein Synthesis Assay Kit (Thermo Fisher Scientific) according to the manufacturer’s instructions.

### Myeloperoxidase ELISA

Fecal samples were collected daily from *S.* Typhimurium-infected mice, immediately frozen in liquid nitrogen, and stored at −80 °C. The samples were suspended at 100 mg/mL in 5 mM EDTA buffer containing 1 mM Tris (pH 7.5), 200 mM NaCl, 1% protease inhibitor cocktail (Sigma-Aldrich), and PhosStop phosphatase inhibitor (1 tablet/10 mL) (Roche). The samples were vortexed for 10 min, then centrifuged at 1500 × *g* for 15 min at 4 °C. Myeloperoxidase levels in the supernatant were measured using a myeloperoxidase ELISA kit (Hycult Biotech Inc.).

### Flow cytometric analysis for murine immune cells

Flow cytometric analysis for immune cells was performed as previously described (17, 18) with modification. For preparation of immune cells, the Peyer’s patches were removed from the intestines of *S.* Typhimurium–infected mice, the intestines were opened longitudinally, washed thoroughly with ice-cold PBS, and cut into 2-cm pieces. The tissue was incubated in 0.5 mM EDTA at 37 °C for 15 min to remove intestinal epithelial cells and intraepithelial lymphocytes. The tissue was further fragmented with scissors, and the fragments were incubated twice at 37 °C for 15 min each in RPMI1640 medium (Sigma-Aldrich) containing 2% newborn calf serum (Equitech-Bio) and 0.5 mg/mL collagenase (Wako Chemicals). The cell suspension was filtered through a 100-μm cell strainer (Corning), centrifuged in a density gradient (820 × *g*, 20 min, 20 °C), and the intestinal mucosal layer lymphocytes were recovered between 40% and 75% Percoll (Cytiva). The cells were stained with anti-CD16/32 monoclonal antibody (TruStain fcX; BioLegend) to prevent nonspecific staining, together with 7-AAD (BioLegend) to detect dead cells; each staining was for 15 min at 25 °C. Neutrophils in the intestinal mucosal layer were stained with FITC anti-Ly6G (1:100; 127606, BioLegend), APC-anti-CD45 (1:100; 103112, BioLegend), and APC-Cy7-anti-CD11b (clone: M1/70; BioLegend) at 4 °C for 30 min. Macrophages derived from bone marrow were stained with PE-anti-I-A^d^ (1:100; 116408, BioLegend), APC-Cy7-anti-CD11b (1:100; 101226, BioLegend), PE-Cy7-anti-F4/80 (1:100; 123114, BioLegend), and BV421-anti-CD45 (1:5; 103133, BioLegend) at 4 °C for 30 min. Cells were washed with 2% newborn calf serum–PBS, and analyzed using the MACSQuant flow cytometer (Miltenyi Biotec) or the CytoFlex LX flow cytometer (Beckman Coulter). The data were analyzed using the FlowJo 9.9 software (Tree Star).

### Stimulated emission depletion microscopy analysis

*S.* Typhimurium was cultured aerobically at 37 °C overnight in LB broth and collected by centrifugation at 10,000 × *g* for 5 min at 25 °C. The bacterial cells (10^8^ CFU) were incubated with 1 µg of L2-1H11 monoclonal antibody dissolved in PBS for 1 h at 37 °C. The cells were washed three times with PBS and incubated with 1 mg/mL Abberior Star Red, goat anti mouse IgG (Abberior) for 1 h at 4 °C. The cells were stained with FM4-64 (Thermo Fisher Scientific), washed three times with PBS and fixed with 4% paraformaldehyde. The samples were analyzed by stimulated emission depletion microscopy under an Abberior Stedycon super-resolution microscope.

### Flow cytometric analysis for bacterial cells

*S.* Enteritidis, Typhimurium, Infantis, Thompson, and Schwarzengrund were cultured aerobically at 37 °C overnight in LB broth and collected by centrifugation at 10,000 × *g* for 5 min at 25 °C. The bacterial cells (10^8^ CFU) were incubated with 1 µg of L2-1H11 antibody dissolved in PBS for 1 h at 37 °C. After the incubation, the cells were washed 3 times with PBS and incubated with APC-labeled anti-mouse IgG (1:100, 405308; BioLegend) for 30 min at 4 °C. The stained bacterial cells were washed three times with PBS and fixed with 4% paraformaldehyde (Nacalai Tesque). The stained samples were analyzed by using a MACSQuant analyzer (Miltenyi Biotech) and data analysis was performed by using the FlowJo 9.9 software (Tree Star).

### Histologic analysis

Frozen tissue was analyzed histologically as described previously with some modifications (17, 18). Tissue samples were washed with PBS on ice and frozen in Tissue-TeK OCT compound (Sakura Finetek) in liquid nitrogen. Frozen tissue sections (6 µm) were prepared by using a cryostat (model CM3050 S) and were fixed in prechilled 95% ethanol (Nacalai Tesque) for 30 min at 4 °C, followed by prechilled 100% acetone (Nacalai Tesque) for 1 min at 25 °C.

For immune-histological analysis, tissue sections were washed twice with PBS for 5 min and blocked in 2% (vol/vol) newborn calf serum in PBS for 30 min at 25 °C in an incubation chamber (Cosmo Bio). Tissue sections were incubated with AF647-labeled anti-Ly6G monoclonal antibody (clone 1A8; 1:100; BioLegend) and FITC-labeled anti-EpCAM monoclonal antibody (clone G8.8; 1:100; BioLegend) in 2% (vol/vol) newborn calf serum in PBS for 16 h at 4 °C; washed once for 5 min each time with 0.1% (vol/vol) Tween-20 (Nacalai Tesque) in PBS and with PBS only; and then stained with DAPI (1 µM; AAT Bioquest) for 10 min at 25 °C in the incubation chamber. Tissue sections were washed twice for 5 min with PBS, mounted in Fluoromount (Diagnostic BioSystems), and examined under a fluorescence microscope (model BZ-9000; Keyence).

### Live/dead staining

*S.* Typhimurium was cultured aerobically at 37 °C overnight in LB broth and centrifuged at 10,000 × *g* for 5 min at 25 °C. The bacterial cells (10^8^ CFU) were incubated with 1 µg of L2-1H11 monoclonal antibody diluted with PBS for 1 h at 37 °C. The cells were washed 2 times with PBS, stained using a Live/Dead BacLight Bacterial Viability Kit (Invitrogen), and washed twice with PBS. The cells were analyzed using MACSQuant, and the data were analyzed using FlowJo 9.9.

### Epitope mapping

The sequence of periplasmic OppA (UniProt ID P06202) from *S.* Typhimurium was elongated with neutral GSGSGSG linkers at the C- and N-termini to avoid truncated peptides and converted into linear sequences of 15 amino acid peptides with a peptide–peptide overlap of 14 amino acids. The resulting OppA peptide microarrays contained 543 different peptides printed in duplicate (1086 spots) and were framed by additional HA (YPYDVPDYAG, 86 spots) control peptides.

### Surface plasmon resonance assay

Using a Biacore T200 (Cytiva), the affinity of antibodies was measured by surface plasmon resonance. Anti-mouse IgG antibodies were immobilized on a CM5 chip using the Mouse Antibody Capture Kit (Cytiva), and L2-1H11 monoclonal antibodies were captured. The measurements were performed at 25 °C in running buffer (PBS supplemented with 0.005% Tween 20). *S.* Typhimurium-derived recombinant OppA was injected at a flow rate of 30 μL/min (association phase), and then running buffer was introduced into the sensor chip (dissociation phase). The association rate constant (K_a_), dissociation rate constant (K_d_), and equilibrium dissociation constant (K_D_) were calculated using BiaEvaluation software (v3.1; cytiva).

### *In silico* analysis

Three-dimensional structure of the protein was predicted by using an open-source software, ColabFold v1.5.5: AlphaFold2 using MMseqs2.1 In accordance with their guidance (19), amino acid sequence was submitted as a query sequence and the three-dimensional structure was predicted and displayed using default parameters.

To analyze the homology of OppA in various serotypes of *Salmonella* and *E. coli*, Multiple Sequence Alignment by CLUSTALW (ver 2.1) was used.

### Metabolome analysis by liquid chromatography–tandem mass spectrometry

Hydrophilic metabolites were extracted as previously described (20). *S.* Typhimurium or *E. coli* DH5α-cultured medium (100 µL) was diluted with water (Wako Pure Chemicals) to a total volume of 200 µL, mixed with 400 µL of methanol (Wako Pure Chemicals) containing methionine sulfone (Wako Pure Chemicals) as an internal standard, and combined with 400 µL of chloroform (Nacalai Tesque). Samples were centrifuged at 20,000 × *g* at 4 °C for 15 min, and 200 µL of supernatant was centrifugally filtered through a 5-kDa cutoff filter (Human Metabolome Technologies). The filtrates were lyophilized, resuspended in ultrapure water (Wako Pure Chemicals), and analyzed by liquid chromatography–tandem mass spectrometry by using a Nexera system (Shimadzu) equipped with two LC-40D pumps, a DGU-405 degasser, an SIL-40C autosampler, a CTO-40C column oven, and a CBM-40 control module and coupled to an LCMS-8050 triple-quadrupole mass spectrometer (Shimadzu). A pentafluorophenylpropyl column (Discovery HS F5, 150 mm × 2.1 mm, 3 µm; Sigma-Aldrich) was used for the separation of metabolites. Instrument control and data analysis were performed by using the software LabSolutions LC-MS with LC-MS/MS Method Package for Primary Metabolites, ver. 2 (Shimadzu). The data analysis was performed using LabSolutions (v.5.120; Shimadzu) and corrected for bacterial number (CFU).

### Subcutaneous immunization

Female BALB/c mice (age, 8 weeks) were subcutaneously immunized with PBS (mock, vehicle control) or *S.* Typhimurium-derived recombinant OppA (10 μg/dose) together with Sigma Adjuvant System (Sigma Aldrich) once a week for 2 consecutive weeks. Serum was collected 1 week after the final immunization. ELISA was performed as described above. Briefly, 96-well immunoplates (Thermo Fisher Scientific) were coated with lyophilized heat-killed *S.* Enteritidis, Typhimurium, Infantis, Thompson, Schwarzengrund (0.1 mg/well) or *S.* Typhimurium-derived recombinant OppA (0.5 µg/well) diluted with PBS at 4 °C overnight and then blocked with 1% (w/v) bovine serum albumin in PBS. Serial dilutions of serum were added to the wells and the plates were incubated for 2 h at 25 °C. The plates were incubated with goat anti-mouse IgG conjugated with horseradish peroxidase and tetramethylbenzidine peroxidase substrate, and the absorbance was measured at 450 nm.

### Oral immunization

Female BALB/c mice (age, 8 weeks) were fasted (>4h~) and orally administered 100 µl of 10% sodium bicarbonate dissolved in ultrapure water to neutralize gastric acid. After 15–20 min, the mice were orally immunized with PBS (mock, vehicle control) or the mixture of *S.* Typhimurium-derived recombinant OppA (10 μg/dose) and Cholera toxin (1 µg/dose, List Biological Laboratories) once a week for 4 weeks. One week after the third or final immunization, serum and feces were collected. Feces were suspended in PBS (100 mg/mL) and vortexed for 10 min at 4 °C. The samples were centrifuged at 3,000 × *g* for 10 min and the supernatants were collected. ELISA was performed as described above. Briefly, 96-well immunoplates (Thermo Fisher Scientific) were coated with lyophilized heat-killed *S.* Enteritidis, Typhimurium, Infantis, Thompson, Schwarzengrund (0.1 mg/well) or *S.* Typhimurium-derived recombinant OppA (0.5µg/well) diluted with PBS at 4 °C overnight and then blocked with 1% (w/v) bovine serum albumin in PBS. Serial dilutions of serum or fecal suspensions were added to the wells and the plates were incubated for 2 h at 25 °C. The plates were incubated with goat anti-mouse IgG or IgA conjugated with horseradish peroxidase and tetramethylbenzidine peroxidase substrate, and the absorbance was measured at 450 nm.

### Survival test in vaccinated mice

Ten days after the final subcutaneous or oral immunization with *S.* Typhimurium- derived recombinant OppA, mice were orally challenged with *S.* Typhimurium as described above. Survival and body weight were monitored daily for 14 days following infection.

### Alanine transaminase and aspartate aminotransferase ELISA

Serum was collected at 96 h post-infection of *S.* Typhimurium. Liver function was evaluated by measuring serum alanine transaminase (ALT) and aspartate aminotransferase (AST) activity by using ALT colorimetric activity assay kit (#700260) and AST colorimetric activity assay kit (#701640) (Cayman Chemical) according to the manufacturer’s instruction.

### Measurement of inflammatory cytokines and chemokines

Serum was collected at 96 h post-infection of *S.* Typhimurium. Systemic inflammatory response against *S.* Typhimurium infection was evaluated by using CBA Mouse Inflammation Kit (BD Biosciences) according to the manufacturer’s instructions.

### Measurement of *Salmonella* turbidity

*S.* Typhimurium was suspended in LB broth or M9 minimal medium (Becton, Dickinson and Company) typically supplemented with 0.2%–0.4% glucose and cultured at 37 °C with shaking at 200 rpm. The absorbance at 600 nm (OD_600_) was measured in a spectrophotometer, and the cells were collected when the OD_600_ reached 0.4–0.6. The cells were then suspended in PBS to an OD_600_ of 0.1. The L2-1H11 antibody (100 µg), Ultra-LEAF™ Purified Mouse IgG1, κ Isotype Ctrl Antibody (100 µg), anti-*S.* Typhimurium LPS antibody (100 µg) or serum derived from OppA-immunized mice (100 µl) was added, and the mixture was incubated at 37 °C for 1 h. LB broth or M9 minimal medium typically supplemented with 0.2%–0.4% glucose was added, and samples were collected at 0, 1, 2, 4, 6, 8, 16, and 24 h, and their OD_600_ was determined by a spectrophotometer.

### Flux analysis

*S.* Enteritidis or Typhimurium was cultured in LB broth or M9 minimal medium typically supplemented with 0.2%–0.4% glucose at 37 °C and 200 rpm/min until 1.0 × 10^8^ CFU/mL. Culture medium was centrifuged 13, 040 × *g* for 3 min at 25 °C. The pellet was diluted in PBS and incubated with 100 µg of L2-1H11 antibody or Ultra-LEAF™ Purified Mouse IgG1, κ Isotype Ctrl Antibody at 37 °C for 1 h. Subsequently, the suspension was seeded into Seahorse 24-well plates (Agilent Technologies) at 100 µL/well coated with 15 µg/mL of Cell-Tak (Corning), followed by centrifugation at 1400 × *g* and 25 °C. LB broth or M9 minimal medium typically supplemented with 0.2%–0.4% glucose, preheated to 37 °C, was added at 400 µL/well, and oxygen consumption rate was measured using a Seahorse Bioscience XF24 Extracellular Flux Analyzer (Agilent Technologies). During the assay, L2-1H11 antibody, serum or fecal suspension derived from OppA-immunized mice was administered 23 minutes after the start. Data were analyzed using the Xfe Wave software (version 2.4.2, Agilent Technologies).

### CFU count

Fecal samples were collected daily from *S.* Typhimurium-infected mice, suspended in LB broth to 100 mg/mL, vortexed for 10 min at 4 °C, and plated onto *Salmonella-Shigella* agar. Plates were incubated overnight at 37 °C, and the number of colonies was counted. Liver and spleen were collected from OppA-immunized mice at 96 h post infection with *S.* Typhimurium, homogenized in LB broth, vortexed, and plated onto *Salmonella-Shigella* agar. Plates were incubated overnight at 37 °C, and the number of colonies was counted.

### Statistical analysis

All line graphs are presented as the mean ± SD. Statistical significance was evaluated by one-way ANOVA for comparison of multiple groups and the Two-tailed student’s *t*-test for two groups by using Prism 10 (GraphPad Software). A *p* value less than 0.05 was considered to be significant.

In general, comparisons between two groups were performed using a two-tailed Student’s t-test, Welch’s t-test, or Mann-Whitney U test, as appropriate. Comparisons among three or more groups were analyzed using one-way ANOVA or two-way ANOVA followed by Tukey’s multiple-comparison test. Time-course experiments involving repeated measurements were analyzed using repeated-measures two-way ANOVA. Survival curves were generated using the Kaplan-Meier method and compared using the log-rank (Mantel-Cox) test. A P value < 0.05 was considered statistically significant.

## Results

### Establishment of monoclonal antibodies with cross-reactivity to multiple *Salmonella* serotypes

To generate monoclonal antibodies with cross-reactivity to multiple *Salmonella* serotypes, we immunized mice subcutaneously with heat-killed whole-cell preparations of *S*. Enteritidis and *S.* Typhimurium, which are the prominent causative agents of *Salmonella*-induced foodborne illnesses in humans due to their marked pathogenicity. We confirmed the presence of antibodies against both *Salmonella* serotypes in the bloodstream (**Supplementary Figure S1**). We then harvested the spleens and lymph nodes, established hybridomas, and determined the specificity of the antibodies produced by each hybridoma by enzyme-linked immunosorbent assay (ELISA). Our analysis revealed that L2-1H11antibody had marked reactivity towards the five major serotypes associated with *Salmonella*-induced food poisoning (*S.* Enteritidis, Typhimurium, Infantis, Thompson, and Schwarzengrund) but very little reactivity toward other commensal or pathogenic bacteria (**Figure 1A**).

**Figure 1:**
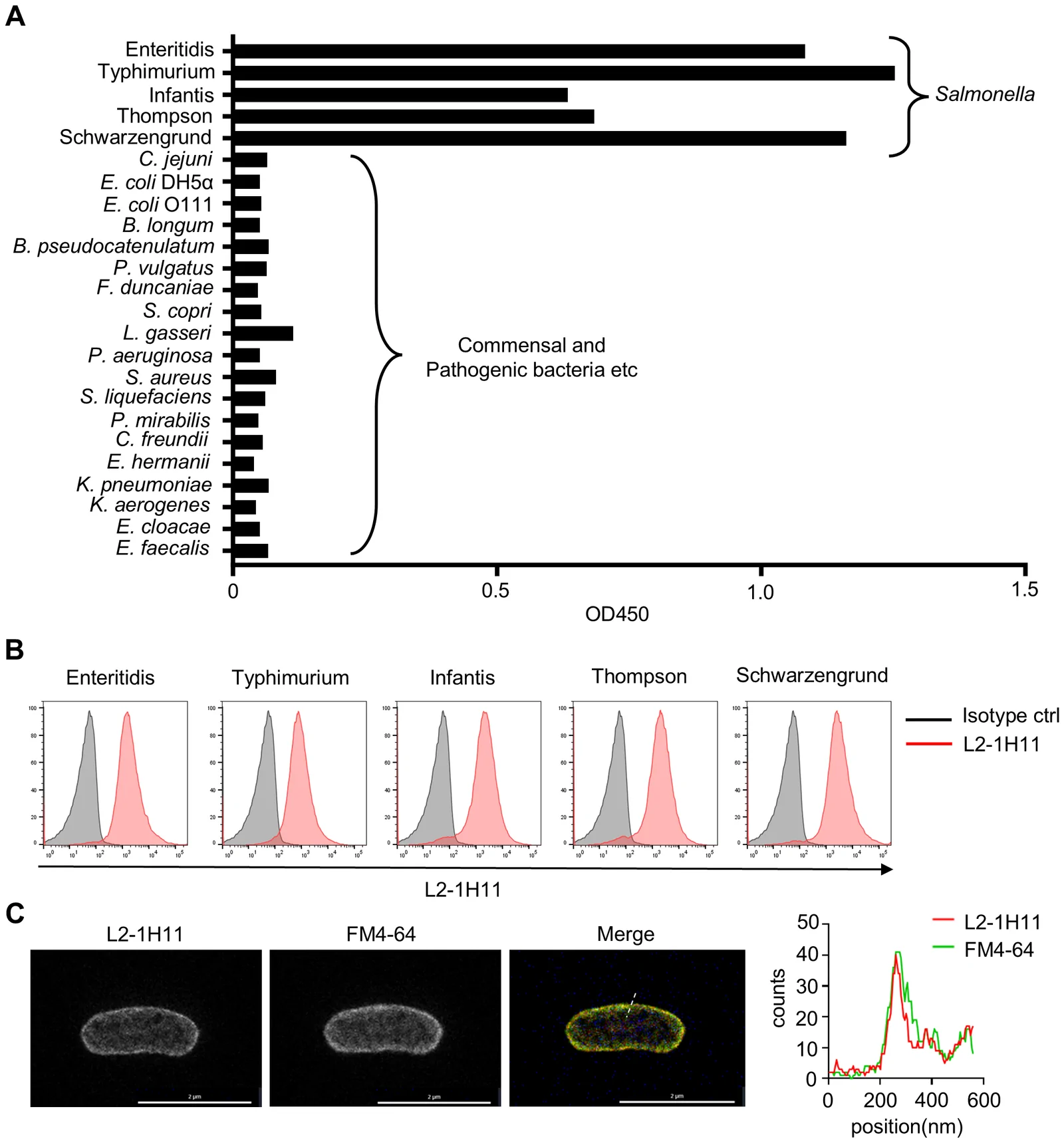
Identification of a cross-reactive monoclonal antibody against various serotypes of *Salmonella*. **(A)** Enzyme-linked immunosorbent assay was used to screen hybridomas for reactivity to a range of heat-killed bacteria, including five Salmonella serotypes. Approximately 2000 clones were tested, and representative data from selected clone L2-1H11 are shown. **(B)** Reactivity of purified L2-1H11 monoclonal antibody to the indicated *Salmonella* serotypes, as determined by flow cytometry. **(C)** Stimulated emission depletion microscopy images and intensity (regions indicated by white line) of *Salmonella Typhimurium* stained with L2-1H11 monoclonal antibody and FM4-64, which labels the bacterial cell surface.

Using fluorescence-activated cell sorting analysis, we confirmed that L2-1H11 reacted with the five major *Salmonella* serotypes (**Figure 1B**). The reactivity of L2-1H11 could be assessed by fluorescence-activated cell sorting analysis, indicating that an L2-1H11-reactive antigen are accessible under the experimental conditions. Super-resolution microscopy revealed distinct signals from the L2-1H11 antibody on the intact *S.* Typhimurium cells (**Figure 1C**). Collectively, these findings support the presence of L2-1H11-reactive antigen that is accessible on intact cells, but they do not establish that native subcellular localization of OppA.

### L2-1H11 antibody protects against *Salmonella* infection by inhibiting bacterial growth

We evaluated the effects of L2-1H11 on critical processes in the establishment of *Salmonella* infection, including adhesion to, invasion of, and induction of cell death in intestinal epithelial cells. In the human epithelial cell line HT29, cell death was significantly lower in the presence of L2-1H11–bound *Salmonella* than in the presence of *Salmonella* with an isotype control antibody (**Figure 2A**). Consistent with these findings, the numbers of bacteria associating to (**Figure 2B**) and invading (**Figure 2C**) HT29 cells were also significantly decreased by binding of the L2-1H11 antibody.

**Figure 2:**
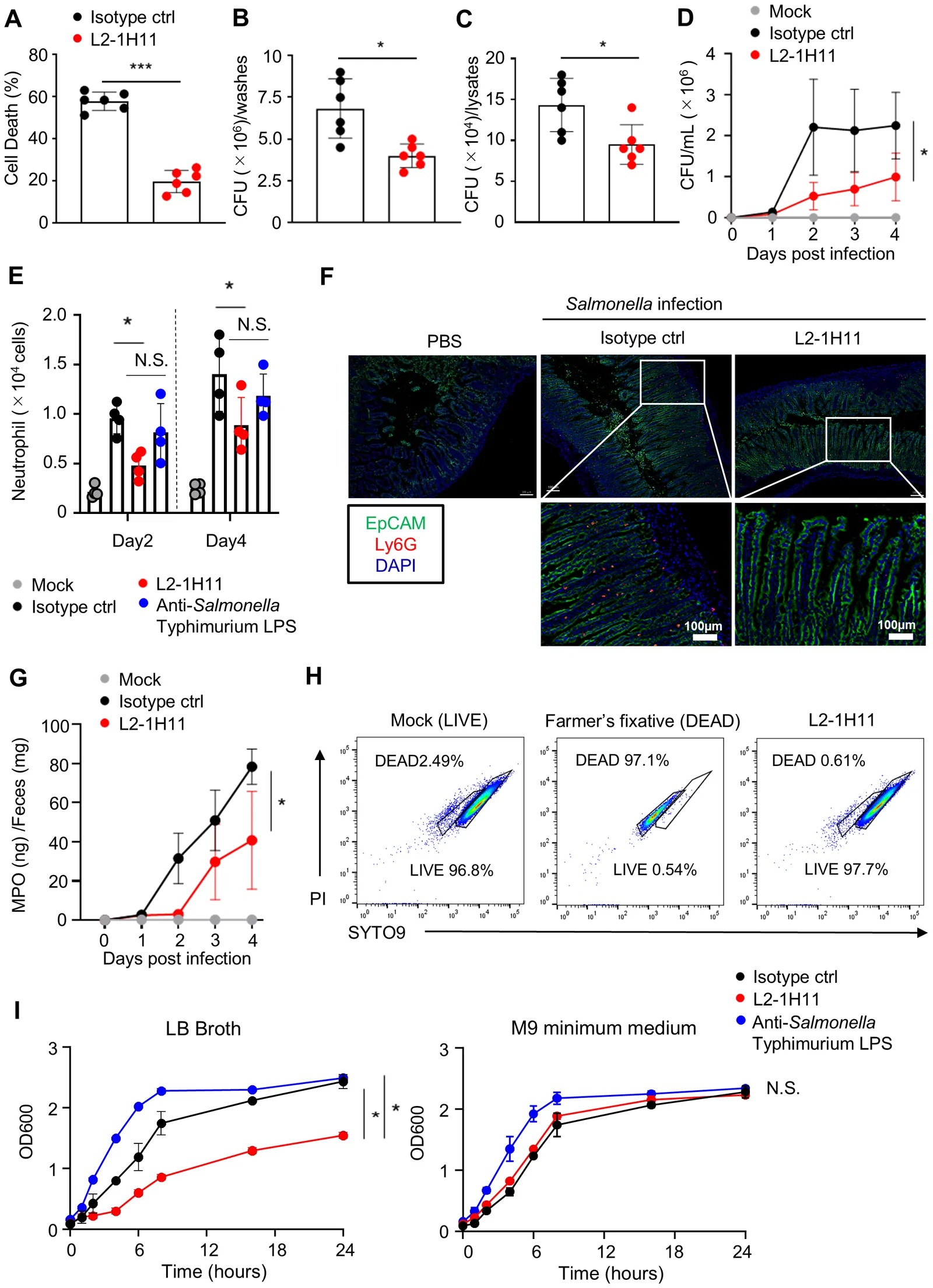
L2-1H11 antibody protects against *Salmonella* infection. **(A)** *Salmonella* Typhimurium cells were treated with 100 μg of isotype control or L2-1H11 antibody, incubated at 37°C for 1 h, and used to infect HT29 cells. Cell death was quantified on the basis of the amount of lactate dehydrogenase released into the culture supernatant (n = 6). OD, optical density. **(B, C)** After adhesion and invasion of HT29 cells by *Salmonella*, the HT29 cells were washed, lysed, and the lysates were plated on *Salmonella*-Shigella agar and cultured overnight. The numbers of colony-forming units (CFUs) were counted (n = 6). **(D)** *Salmonella* Typhimurium (1.0 × 106 CFU) was treated with 100 μg of isotype control or L2-1H11 antibody and incubated at 37°C for 1 h. Mice were orally infected with the treated bacteria using a gavage tube. Fecal samples were collected daily, suspended in LB broth, plated on Salmonella-Shigella agar, incubated overnight, and the number of CFUs was counted. **(E)** The intestines were excised 2 and 4 days post-infection with *Salmonella* Typhimurium conjugated with L2-1H11 antibody or anti-*Salmonella* Typhimurium LPS antibody, and neutrophils in the lamina propria were counted on the basis of flow cytometric analysis data. **(F)** Immunohistological analysis of the ileum 4 days post-infection. Neutrophils, epithelial cells, and nuclei were stained with Ly6G monoclonal antibody (red), EpCAM monoclonal antibody (green), and DAPI (blue), respectively. **(G)** Fecal samples from infected mice were collected daily, and the concentration of myeloperoxidase (MPO), which is specifically secreted by neutrophils, was quantified by enzyme-linked immunosorbent assay. **(H)** *Salmonella* Typhimurium cells were treated with 1 μg of isotype control or L2-1H11 antibody and incubated at 37°C for 1 h. They were then stained by using a Live/Dead BacLight Bacterial Viability Kit, and the ratio of live to dead *Salmonella* was determined by using a flow cytometer. **(I)** *Salmonella* Typhimurium cells were treated with 100 μg of isotype control, L2-1H11 antibody, or anti-*Salmonella* Typhimurium LPS antibody and incubated at 37°C for 1 h, transferred to LB broth or M9 minimal medium, and bacterial growth was assessed at 0, 1, 2, 4, 6, 8, 16, and 24 h. Data are representative of two independent experiments and are presented as mean ± SD. Two-tailed Student’s t-test **(A–C)**, one-way ANOVA **(E)**, two-way ANOVA **(I)** and repeated-measures two-way ANOVA **(D, G)**: *P < 0.05, ***P < 0.001. Scale bars, 100 μm.

Next, we assessed the efficacy of L2-1H11 in an *in vivo* infection model. To mimic the *in vivo* situation in which antigen-specific antibodies are present in the intestinal lumen and directly bind bacteria prior to epithelial invasion, we orally infected mice with *Salmonella* coated with L2-1H11. In these mice, the number of bacteria in the feces was significantly reduced in comparison with an isotype control (**Figure 2D**). *Salmonella* enterocolitis typically entails the infiltration of neutrophils into the intestinal mucosa. However, when mice were orally infected with L2-1H11–bound *Salmonella*, minimal accumulation of neutrophils was observed in the lamina propria of the small intestine (**Figure 2E**), as evidenced by histological analysis (**Figure 2F**). Notably, neutrophil numbers were lower in mice infected with L2-1H11–bound bacteria than in those infected with bacteria treated with anti-*S.* Typhimurium LPS antibody (**Figure 2E**). Furthermore, the fecal myeloperoxidase, a bactericidal molecule secreted by neutrophils, was reduced in the L2-1H11 antibody group (**Figure 2G**). Given the possibility that antibody-mediated opsonization might enhance phagocytic clearance, we further examined this using bone marrow–derived macrophages. Compared with the isotype control, treatment with either L2-1H11 or anti-*S.* Typhimurium LPS resulted in a significant increase (**Supplementary Figure S2A**). To directly assess antibody-mediated protection, we performed passive transfer experiments. Intravenous administration of the purified L2-1H11 antibody prior to infection significantly suppressed body weight loss and improved survival (**Supplementary Figure S2B**). These results suggest that protection is largely antibody-dependent.

Having observed efficacy against *Salmonella* infection both *in vitro* and *in vivo*, we proceeded to investigate the direct effects of L2-1H11 antibody on *Salmonella* cells. Analysis using Live/Dead staining did not reveal any bactericidal effects of L2-1H11 antibody (**Figure 2H**). However, L2-1H11–treated *Salmonella* showed reduced growth compared with isotype control and anti-LPS antibody–treated groups, particularly during the exponential phase in LB broth (**Figure 2I**). To test the OppA-dependence hypothesis, we performed the same experiment in M9 minimal medium, which lacks amino acids. The growth-inhibitory effect of L2-1H11 was abolished, and bacteria grew similarly to those in isotype control and anti-LPS groups (**Figure 2I**). When we used qRT-PCR to examine the changes in the expression of genes encoding molecules involved in *Salmonella* adhesion, invasion, and motility, we did not observe any changes following the addition of L2-1H11 (**Supplementary Figure S2C–E)**.

Thus, the L2-1H11 antibody appears to inhibit the growth of *Salmonella* through a bacteriostatic effect, thereby reducing damage to the intestinal epithelium. This inhibition may impede the pathogenesis of *Salmonella* infections, including enteritis, by preventing damage to the intestinal epithelium.

### OppA is the candidate antigen recognized by L2-1H11 antibody

Our experiments described above demonstrated that L2-1H11 is a functional antibody that can inhibit *Salmonella* growth and contribute to the suppression of enteritis. Next, to identify the target molecule of L2-1H11, the molecule binding to the antibody was recovered by immunoprecipitation and subjected to proteomic analysis using mass spectrometry. As a result, OppA was identified as a candidate molecule recognized by L2-1H11 (**Figures 3A, B**; **Supplementary Table 1**). To confirm this finding, we produced a recombinant OppA protein and found that it too reacted with L2-1H11 (**Figure 3C**). Characterization by surface plasmon resonance assay revealed that L2-1H11 had a high binding affinity for OppA (*K*_D_ = 4.211 × 10^−11^ M; **Figure 3D**).

**Figure 3:**
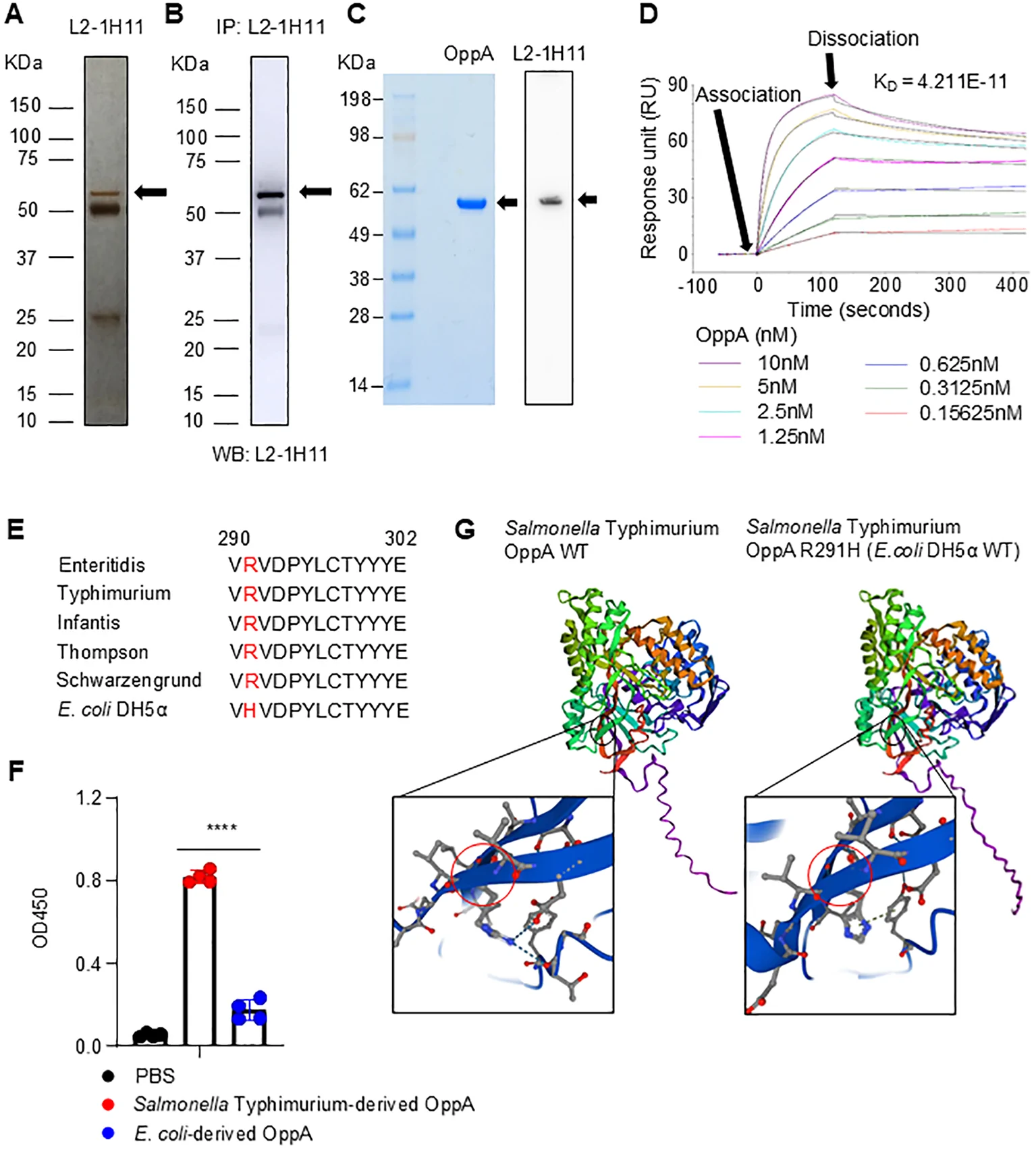
L2-1H11 antibody recognizes oligopeptide-binding protein A (OppA). Immunoprecipitation **(A)** and western blot analysis **(B)** of a lysate of *Salmonella* cultured in LB broth in the presence of L2-1H11 antibody. **(C)** NuPAGE electrophoresis of recombinant OppA protein and western blot analysis using L2-1H11. **(D)** Binding affinity of L2-1H11 to OppA was quantitatively analyzed by surface plasmon resonance assay. **(E)** Comparison of amino acids 290–302 of OppA from the indicated *Salmonella* serotypes and Escherichia coli DH5α. **(F)** L2-1H11 antibody binding to recombinant OppA derived from *Salmonella* Typhimurium or E. coli was evaluated by enzyme-linked immunosorbent assay to assess potential off-target cross-reactivity with commensal bacteria. Absorbance was measured at 450 nm. Data are shown as mean ± SD of two independent experiments. **(G)** Three-dimensional structure of *Salmonella* Typhimurium OppA shown alongside its single-point mutant (R291H), as predicted using the AlphaFold structure prediction tool. The location of the substituted amino acid is indicated by red circles.

Next, we conducted an epitope mapping analysis to determine the L2-1H11 recognition site on OppA. We found that L2-1H11 recognized amino acid residues 290–302 of *S.* Typhimurium-derived OppA. *In silico* analysis using the ClustalW tool to investigate the recognition epitope of L2-1H11 antibody revealed that amino acid residue 291 of OppA differs in *E. coli* compared with the five major serotypes of *Salmonella* (**Figure 3E**, **S3**). We performed ELISA using recombinant *Salmonella* OppA and recombinant *E. coli* OppA proteins. While L2-1H11 showed clear reactivity to *Salmonella* OppA, no detectable binding was observed to *E. coli* OppA (**Figure 3F**). A prediction by the AlphaFold structure prediction tool indicated that the amino acid difference at position 291 may influence the local structure of the identified epitope region (**Figure 3G**).

Together, these results demonstrate preferential recognition of *Salmonella* OppA by L2-1H11 and suggest a possible contribution of residue 291 to antibody binding specificity.

### L2-1H11 antibody is associated with reduced amino acid abundance and suppressed energy metabolism

OppA is a subunit of the oligopeptide transporter Opp, which captures free peptides and transports them into the cell (10, 21). Peptides transported through OppA into the cell are then degraded into amino acids by peptidases and supplied to *Salmonella* as a nutrient source, thereby contributing to protein synthesis, intracellular metabolism, and energy production (22). We therefore examined the levels of amino acids in *Salmonella* cells by liquid chromatography–tandem mass spectrometry and found that in L2-1H11–treated *Salmonella* the concentrations of all of the essential and non-essential amino acids were significantly decreased over time (**Figure 4A**; **Supplementary Figure S4A**), whereas in *E. coli* no such decreases were observed (**Supplementary Figure S5**).

**Figure 4:**
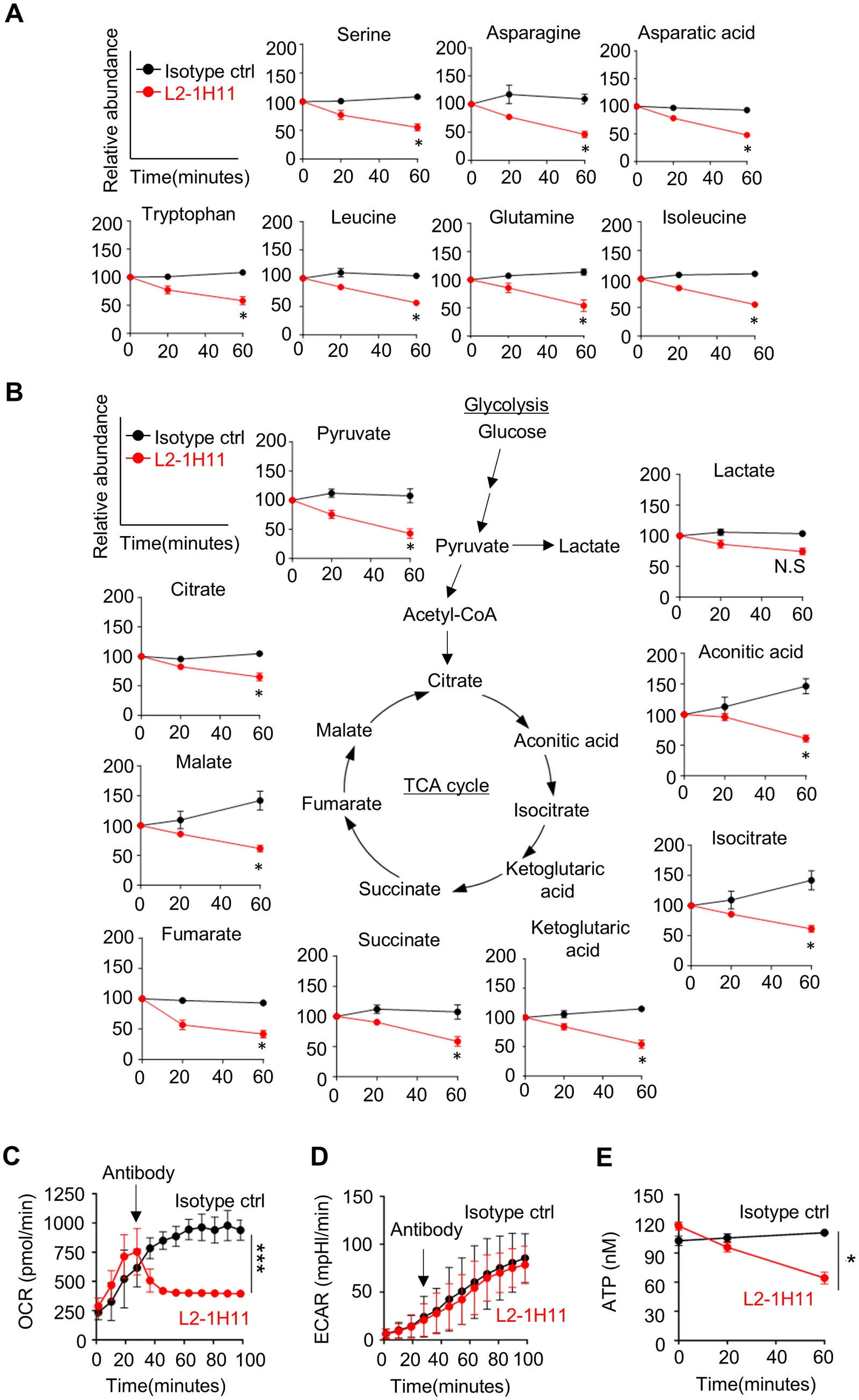
L2-1H11 antibody is associated with reduced intracellular amino acid abundance and suppressed energy metabolism in *Salmonella*. *Salmonella* cells were treated with 100 µg of either isotype control or L2-1H11 antibody. **(A, B)** A time-course analysis of the concentrations of major amino acids **(A)** and key metabolites from glycolysis (lactate and pyruvate) and the tricarboxylic acid (TCA) cycle **(B)** in *Salmonella* cells by liquid chromatography–tandem mass spectrometry (n = 4). **(C, D)** Oxygen consumption rate (OCR) and extracellular acidification rate (ECAR) (n = 4). **(E)** ATP concentration in *Salmonella* cells (n = 4). Data are representative of two independent experiments and are presented as mean ± SD. Two-tailed Student’s t-test: *P < 0.05, ***P < 0.001.

Some intracellular amino acids are utilized for protein synthesis and energy production through the citric acid cycle (23). To investigate the impact of the L2-1H11 antibody on protein synthesis, a pulse–chase assay was conducted, revealing no changes in protein synthesis associated with treatment with L2-1H11 (**Supplementary Figure S6**). Metabolomic analysis of intracellular energy metabolism revealed no changes in *E. coli*, which does not react to L2-1H11 antibody (**Supplementary Figure S7A**), but a decrease in metabolites of the citric acid cycle was observed in *S.* Typhimurium treated with L2-1H11 (**Figure 4B**). Flux analysis showed a significant decrease of oxygen consumption rate, an indicator of bacterial respiratory activity, upon addition of L2-1H11 antibody in *S*. Typhimurium (**Figure 4C**) and in *S.* Enteritidis (**Supplementary Figure S4B**). Conversely, no changes were observed for extracellular acidification rate, an indicator of glycolysis, in *S.* Typhimurium or Enteritidis upon addition of L2-1H11 antibody (**Figure 4D**; **Supplementary Figure S4C**). To address whether the observed metabolic suppression depends on nutrient conditions, we performed flux analyzer experiments in M9 minimal medium. In contrast to LB broth, the suppressive effect of L2-1H11 on metabolic activity (as measured by oxygen consumption rate) was abolished in minimal medium (**Supplementary Figure S4D**). The decrease in oxygen consumption rate in *S.* Typhimurium was accompanied by a significant decrease in intracellular ATP concentration upon treatment with the L2-1H11 antibody (**Figure 4E**). In contrast, in *E. coli* no fluctuations were observed in extracellular acidification rate or oxygen consumption rate, and there was no impact on ATP production (**Supplementary Figure S7B–D**).

Together, these findings indicate that L2-1H11 antibody recognizes OppA and that L2-1H11 treatment is accompanied by reduced intracellular amino acid abundance and suppression of citric acid cycle-associated energy metabolism in *Salmonella* cells. These observations are consistent with, but do not yet prove, interference with OppA-associated functions involved in oligopeptide utilization.

### Induction of acquired immune responses against *Salmonella* and suppression of salmonellosis by using OppA as a systemic or oral vaccine antigen

Our results indicated that L2-1H11 antibody suppresses the pathogenesis of *Salmonella* infection and that OppA is a candidate antigen recognized by this protective antibody, suggesting that OppA may serve as a candidate antigen capable of inducing antibody responses that recognize multiple *Salmonella* serotypes. To investigate this possibility, serum was collected from mice subcutaneously immunized two times with recombinant OppA along with an adjuvant, and the binding ability of this serum to *Salmonella* bacteria was examined by ELISA. In the immunized group, production of antibodies reactive to the five serotypes of *Salmonella* was observed (**Figure 5A**). The serum collected from vaccinated mice suppressed the growth and respiratory activity of *S.* Typhimurium similarly to L2-1H11 antibody (**Figures 5B–D**), indicating that serum obtained from OppA-immunized mice exhibited inhibitory effects on *Salmonella* growth and respiratory activity under this experimental condition.

**Figure 5:**
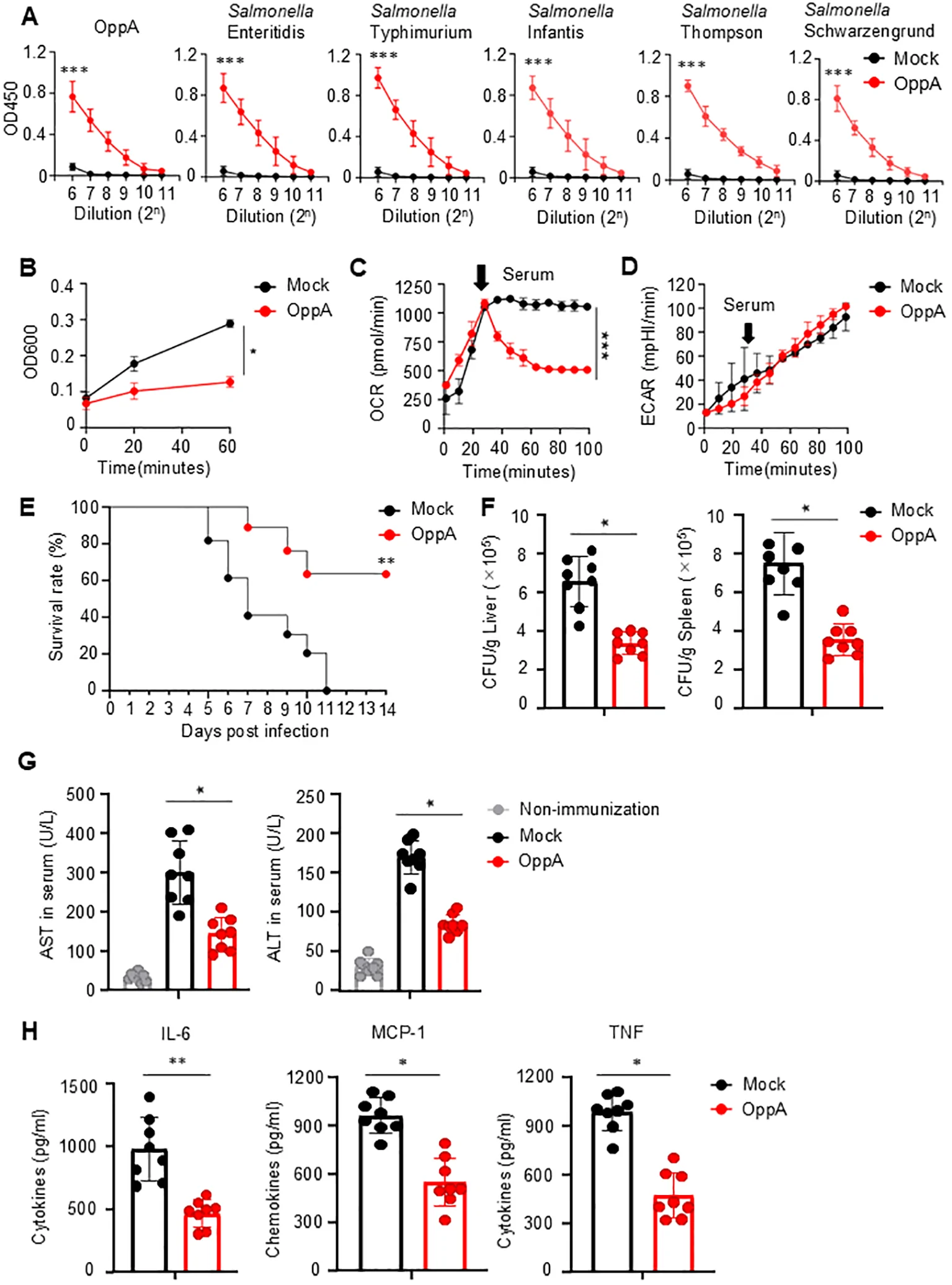
Subcutaneous immunization of mice with OppA induces acquired immune responses against *Salmonella* and suppresses salmonellosis. **(A)** Detection of *Salmonella* serotype–specific serum IgG antibodies by enzyme-linked immunosorbent assay (n = 10) in mice subcutaneously immunized with phosphate-buffered saline (mock) or OppA once a week for 2 consecutive weeks. **(B)** *Salmonella* Typhimurium cells were treated with serum from mice subcutaneously immunized with phosphate-buffered saline (mock) or OppA and incubated at 37°C for 1 h; bacterial growth was subsequently evaluated by measuring the turbidity of *Salmonella* culture (n = 8). **(C, D)** OCR and ECAR in *Salmonella* treated with serum collected from mice subcutaneously immunized with mock or OppA (n = 8). **(E)** Survival rates of mice subcutaneously immunized with mock or OppA were monitored for 2 weeks post–oral infection with *Salmonella* (n = 10). **(F)** Colony-forming units (CFUs) of *Salmonella* in the liver and spleen were counted 4 days post-infection in mice subcutaneously immunized with mock or OppA and orally infected with *Salmonella* (n = 8). **(G)** Enzyme activity levels of alanine transaminase (ALT) and aspartate aminotransferase (AST) in serum were measured on day 4 post-infection in naïve mice (non-immunization) and mice subcutaneously immunized with mock or OppA (n = 8). **(H)** Cytokine or chemokine concentrations in the serum from mice subcutaneously immunized with mock or OppA and orally infected with *Salmonella* were quantified using a Cytometric Bead Array assay (n = 8). IL, interleukin; MCP, monocyte chemoattractant protein; TNF, tumor necrosis factor. Data from two independent experiments were combined and are presented as mean ± SD. Two-tailed Student’s t-test **(A–F, H)**, one-way ANOVA **(G)**, and log-rank (Mantel-Cox) test **(E)**: *P < 0.05, **P < 0.01, ***P < 0.001.

To evaluate the protective efficacy of the OppA-containing vaccine formulation against salmonellosis, mice vaccinated with mock or OppA were orally infected with *S.* Typhimurium, and their survival was examined. In the mock-treated group, the body weight of infected mice decreased over time (**Supplementary Figure S8A**), with individual deaths starting from day 4 post-infection, and by day 14 post-infection almost all infected mice had died (**Figure 5E**; **Supplementary Figure S8A**). In the group vaccinated with the OppA-containing vaccine formulation, survival rate improved and the body weight of surviving mice temporarily decreased but recovered over time (**Figure 5E**; **Supplementary Figure S8A**).

*Salmonella* infection in the intestinal tract is known to manifest as systemic symptoms, followed by dissemination to the liver and spleen (24). Therefore, we evaluated the bacterial counts in feces and the inflammatory state of the small intestine, liver, and spleen after oral infection with *S.* Typhimurium in vaccinated mice. The bacterial counts over time in the intestinal lumen were similar in mice vaccinated with OppA vaccine and in the mock-treated group (**Supplementary Figure S8B**). This was consistent with the observation of neutrophil infiltration and myeloperoxidase production in the feces in both groups (**Supplementary Figure S8C**). On the other hand, in the liver and spleen, mice vaccinated with OppA had significantly fewer bacteria compared to the mock-treated group (**Figure 5F**). Furthermore, OppA-vaccinated mice infected with *S.* Typhimurium showed suppressed elevation of the liver function markers alanine transaminase (ALT) and aspartate aminotransferase (AST) compared to mock-vaccinated mice (**Figure 5G**). In the serum of vaccinated mice, the productions of cytokines and chemokines such as IL-6, tumor necrosis factor, and monocyte chemoattractant protein-1, which are known to increase during *Salmonella* infection, were also significantly suppressed (**Figure 5H**).

The potential utility of OppA as a mucosal vaccine was evaluated in mice orally immunized with OppA. Oral immunization with OppA induced the production of fecal IgA and serum IgG antibodies against OppA as well as against whole *Salmonella* cells, including *S.* Enteritidis, Typhimurium, Infantis, Thompson, and Schwarzengrund (**Figure 6A**, **Supplementary Figure S9A**). Notably, antibody responses—particularly those directed against *Salmonella* bacterial cells—were more strongly induced after four immunizations compared with three immunizations (**Supplementary Figure S9A**). A flux analysis was also conducted to assess the effect of fecal IgA antibodies on *Salmonella* energy metabolism. Although no changes were observed in extracellular acidification rate (**Figure 6B**), Fecal extracts obtained from OppA-immunized mice decreased the oxygen consumption rate of *S*. Typhimurium similarly to subcutaneous immunization (**Figure 6C**). Consistent with this result, the number of *Salmonella* in feces, the cell number of neutrophils in the lamina propria of the small intestine, and the amount of fecal myeloperoxidase were all decreased in *Salmonella*-infected mice following oral administration of OppA vaccine (**Figures 6D–F**). OppA-vaccinated mice showed attenuated weight loss compared to the mock group and recovered to pre-infection weight two weeks post-infection (**Supplementary Figure S9B**), and they also showed an increased survival rate (**Figure 6G**; **Supplementary Figure S9B**). Subsequent evaluation revealed a significant decrease in *Salmonella* counts in the liver and spleen, as well as significantly decreased ALT and AST levels and inflammatory cytokine concentrations in serum of the OppA-vaccinated group, compared to the mock group (**Figures 6H–J**). Thus, these results support the potential utility of OppA as a mucosal vaccine antigen.

**Figure 6:**
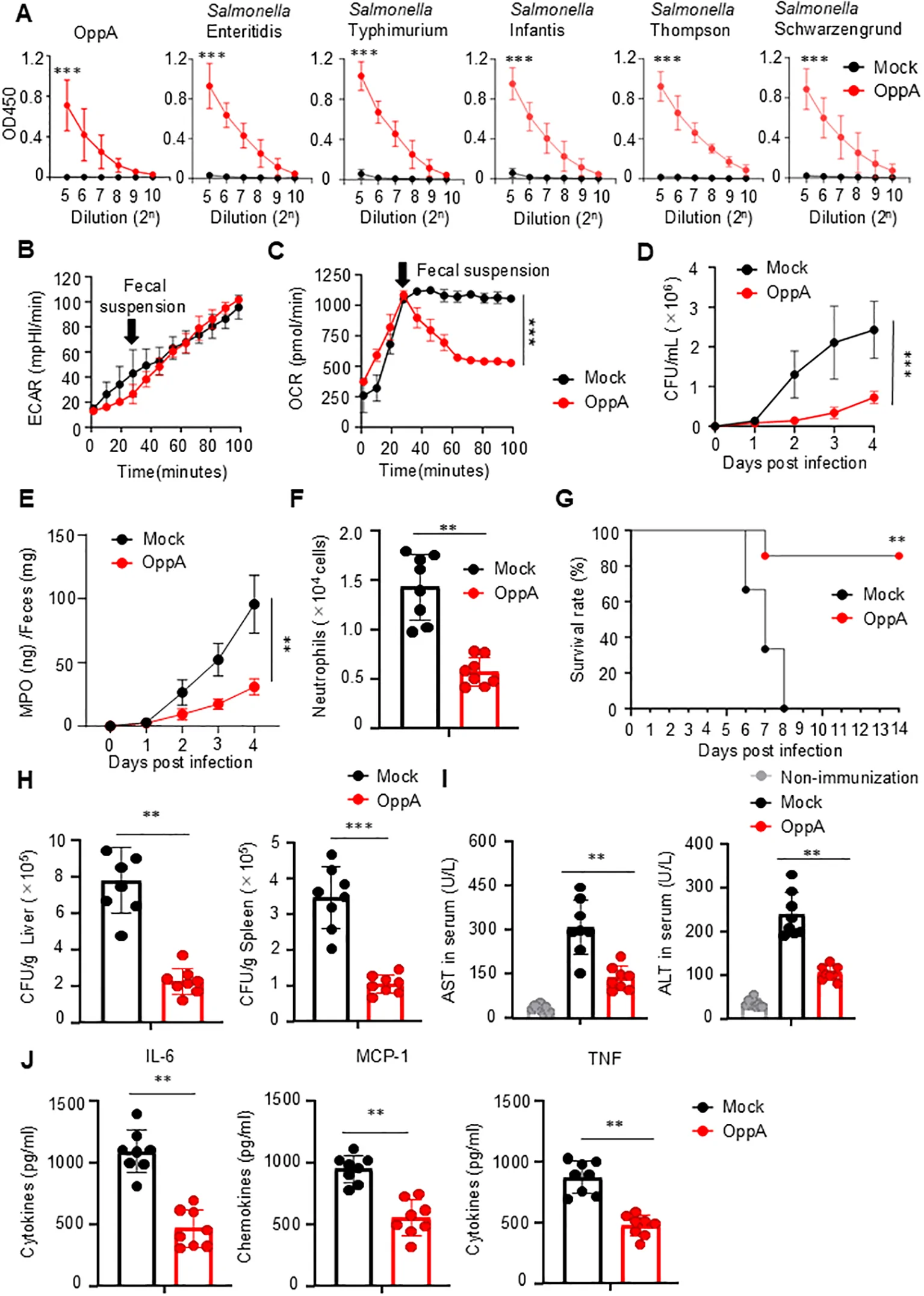
Protective effects and immune responses induced by oral immunization with OppA in mice against salmonellosis. Mice were orally immunized with either phosphate-buffered saline (mock) or OppA once a week for four times. **(A)** One week after the final immunization, a feces sample was collected for the detection of *Salmonella*-specific fecal IgA by enzyme-linked immunosorbent assay (n = 10). **(B, C)** ECAR and OCR were measured in *Salmonella* treated with the fecal suspension collected from mice immunized orally with mock or OppA (n = 4). **(D)** Number of *Salmonella* in feces from immunized with mock or OppA and infected mice was monitored daily. Fecal samples were suspended in LB broth, and the supernatant of the fecal slurry was plated on *Salmonella*-Shigella agar and cultured overnight to count colony-forming units (CFUs). **(E)** Myeloperoxidase (MPO) levels were quantified daily in feces from immunized and infected mice by enzyme-linked immunosorbent assay (n = 4). **(F)** Four days post-infection, the intestines were collected from mice orally immunized with mock or OppA, and the number of neutrophils in the lamina propria was quantified by flow cytometric analysis. **(G)** Survival rates of mice orally immunized with mock or OppA were monitored for 2 weeks post-infection with *Salmonella*. **(H)** CFUs of *Salmonella* in the liver and spleen were assessed on day 4 post-infection in mice that had been orally immunized and then infected with *Salmonella*. **(I)** Serum samples were collected from naïve mice (non-immunization) and mice orally immunized with mock or OppA on day 4 post-infection, and activity of alanine transaminase (ALT) and aspartate aminotransferase (AST) and **(J)** concentrations of cytokines or a chemokine were determined (n = 4). IL, interleukin; MCP, monocyte chemoattractant protein; TNF, tumor necrosis factor. Data are representative of two independent experiments and are presented as mean ± SD. Two-tailed Student’s t-test **(A–G, J)**, one-way ANOVA **(H, I)**, repeated-measures two-way ANOVA **(D, E)**, and log-rank (Mantel-Cox) test **(G)**: **P < 0.01, ***P < 0.001.

## Discussion

In the present study, we identified L2-1H11, a monoclonal antibody that recognizes OppA as a candidate *Salmonella* antigen and is associated with bacteriostatic activity, reduced respiratory activity, and attenuation of *Salmonella* enteritis. Immunizing mice with OppA as a vaccine antigen induced neutralizing antibodies against *Salmonella*, which inhibited the pathogenesis of *Salmonella* enteritis and the dissemination of *Salmonella* to remote organs such as the liver and spleen, increasing the survival rate of mice after *Salmonella* infection. This highlights the potential relevance of OppA as a target for antibody therapeutics and a vaccine antigen against *Salmonella*, while further mechanistic validation remains necessary.

Many treatments for bacterial infections rely on antibiotics, which work by inhibiting processes like bacterial nucleic acid synthesis or cell wall synthesis (25). However, concerns about the emergence of antibiotic-resistant bacteria have prompted the urgent need for alternative therapies (26). Antibody therapeutics have emerged as a promising avenue in this regard (27, 28). Recent research has focused on controlling bacterial enteritis using monoclonal antibodies. For example, monoclonal secretory IgA antibodies specific to FliD from *Campylobacter jejuni* and to CfaE from enterotoxigenic *E. coli* have been reported to prevent infection by inhibiting the motility of *C. jejuni* and the adherence of enterotoxigenic *E. coli* to the intestinal epithelium (29–31). In the case of *Salmonella*, monoclonal secretory IgA antibodies recognizing the O antigen of *S.* Typhimurium LPS can inhibit uptake of *S.* Typhimurium into Peyer’s patches by binding and agglutinating the bacteria (32–38). Similarly, the monoclonal IgG antibody mAb-449, which recognizes the LPS O antigen, can enhance phagocytosis of *Salmonella* by macrophages, contributing to infection defense (9). However, given that these antibodies primarily promote phagocytosis of bacteria by cells such as macrophages and also the vast diversity of *Salmonella* serotypes, we believed that antibodies cross-reactive against multiple *Salmonella* serotypes may provide broader antigen recognition and therefore represent promising candidates for the development of broadly applicable *Salmonella* intervention strategies.

In the present study, we used a method of immunizing rats with mouse immune cells without specifying a target molecule to establish an immune cell–specific antibody library, which allowed us to identify effective antibodies based on their functionality and reactivity (39). We applied this method to *Salmonella*, constructing a *Salmonella*-specific antibody library, and ultimately identified L2-1H11, a monoclonal IgG antibody that was cross-reactive against various serotypes of *Salmonella* (Enteritidis, Typhimurium, Infantis, Thompson, and Schwarzengrund). Approximately 50% of *Salmonella* foodborne outbreaks reported in Japan have been attributed to the five serotypes examined in this study (**Supplementary Table S2**). Together with the global predominance of *S*. Enteritidis and *S*. Typhimurium (40, 41), these findings suggest broad serotype reactivity against epidemiologically important *Salmonella* serotypes. While protective efficacy was demonstrated against *S*. Typhimurium in the present study, cross-protection against other serotypes remains to be determined. However, because more than 2,600 serotypes have been described, broader validation, including additional non-typhoidal *Salmonella* serotypes and *S. Typhi*-related models, will be required to determine the full applicability of L2-1H11.

We believe that the L2-1H11 monoclonal antibody contributes to defense against *Salmonella* infection through bacteriostatic activity. However, the extent to which these effects are directly mediated through OppA remains to be fully clarified. The bacteriostatic activity of L2-1H11 may indirectly reduce intestinal inflammation by limiting bacterial physiological activity and attenuating downstream inflammasome-associated inflammatory responses (42–48). However, the precise mechanisms linking bacterial growth suppression to reduced intestinal pathology remain to be determined. Interestingly, the survival benefit conferred by OppA immunization appeared greater than the reduction in bacterial burden observed in systemic organs (**Figures 5E, F**, **6G, H**), suggesting that suppression of bacterial physiological activity may contribute to protection. Consistent with this interpretation, L2-1H11 reduced bacterial energy metabolism under our experimental conditions. Future transcriptomic, metabolomic, and long-term persistence studies will be required to determine how metabolic suppression influences virulence, bacterial survival, and disease outcome (49).

Starvation of nutrients such as amino acids and sugars, as well as changes in pH and growth temperature, are well-known triggers of bacterial growth arrest. In response to such environmental stresses, bacteria including *Salmonella* reduce DNA replication, transcription, translation, and energy production. One possible explanation for the bacteriostatic effect of L2-1H11 is interference with OppA-associated peptide utilization, leading to reduced intracellular amino acid availability and altered energy metabolism. Consistent with this interpretation, L2-1H11 treatment was associated with decreased intracellular amino acid abundance, ATP production, and respiratory activity. OppA is the substrate-binding component of the conserved Opp ABC transporter and plays an important role in peptide utilization in diverse bacterial species (10, 50–53). Notably, gene expression analysis by qRT-PCR revealed no marked changes in the expression of genes related to protein synthesis, adhesion, invasion, or motility following L2-1H11 treatment. A similar phenomenon has been reported in *E. coli*, where restriction of amino acid-containing nitrogen sources suppresses assimilatory pathways while maintaining expression of protein synthesis-related genes despite reduced growth rates (54). Furthermore, previous studies have demonstrated that perturbations of central carbon metabolism can influence *Salmonella* virulence, motility, intracellular survival, and susceptibility to host immune defenses (55, 56). Together, these findings support the possibility that the metabolic alterations associated with L2-1H11 treatment may contribute not only to suppression of bacterial growth but also to enhanced host defense. However, our findings do not establish OppA as the sole functional target responsible for the observed antibacterial activity. Additional studies, including localization analyses, peptide transport assays, and experiments using *oppA*-deficient mutants, will be required to clarify the contribution of OppA to the biological effects of L2-1H11.

The L2-1H11 antibody may interfere with OppA-associated functions linked to peptide utilization in *Salmonella*, together with suppression of bacterial respiratory activity and energy metabolism observed under our experimental conditions. From the perspective of energy metabolism, competition and mutual utilization between bacteria and host cells are important factors. For example, vitamin B6 produced by gut bacteria of the *Bacteroides* genus (e.g., *B. acidifaciens*) contributes to the clearance of *S.* Typhimurium in the intestinal tract and suppression of enteritis (57). Short-chain fatty acids such as acetate, propionate, and lactate produced by gut bacteria bind to the adapter protein ASC in the inflammasome pathway, inducing inflammasome activation including IL-1β production, and inhibition of *S.* Typhimurium growth in macrophages (58).

We also found that immunization with an OppA-containing vaccine formulation induced antibody responses with functional properties similar to L2-1H11. OppA may represent a complementary vaccine target distinct from conventional LPS- or flagellin-based antigens because antibodies induced by OppA vaccination showed reactivity against multiple *Salmonella* serotypes evaluated in this study, and OppA is associated with bacterial metabolic fitness. Although the contribution of OppA itself remains to be established, these characteristics support its potential inclusion in multicomponent vaccines designed to provide broad serotype coverage and complementary mechanisms of protection. OppA has also been investigated as a vaccine antigen in *Moraxella catarrhalis*, where OppA-specific antibodies reduce bacterial colonization (59–61). Together with our findings, these observations support the broader potential of OppA-based vaccination strategies. In the present study, OppA immunization induced OppA-reactive immune responses and was associated with reduced *Salmonella* dissemination and improved survival, although the precise protective mechanism remains to be fully elucidated. Whether mucosal OppA-specific IgA responses contribute to intestinal protection remains an important question for future investigation.

Importantly, we evaluated the potential risk of off-target effects, including cross-reactivity with commensal bacteria such as *E. coli*. No detectable antibody cross-reactivity against recombinant OppA derived from *E. coli* was observed in ELISA (**Figure 3F**), indicating a low likelihood of unintended recognition of *E. coli* OppA. However, this observation was based on the monoclonal antibody L2-1H11 and should not be interpreted as a comprehensive assessment of the polyclonal antibody response elicited by recombinant OppA vaccination. Immunization with full-length OppA is expected to induce antibodies recognizing multiple epitopes across the protein, and the resulting specificity profile may vary among host genetic backgrounds. Further studies using polyclonal immune sera and broader bacterial panels will therefore be required to comprehensively evaluate potential cross-reactivity and off-target effects. These findings suggest that the specificity of the induced antibody response is preserved, and that disruption of commensal bacterial populations may be limited. Several limitations should nevertheless be acknowledged. A limitation of the present vaccination study is that adjuvant-only control groups were not included. Therefore, the relative contribution of OppA and the adjuvants used (Sigma Adjuvant System and cholera toxin) to the observed protective effects could not be determined. Future studies using adjuvant-only controls will be required to define the antigen-specific contribution of OppA to vaccine-induced protection. In addition, residual endotoxin levels in recombinant OppA preparations were not directly measured. Therefore, the contribution of trace endotoxin contamination to immune activation cannot be completely excluded. Furthermore, the present study employed a streptomycin-pretreated mouse model; therefore, validation in human microbiota-associated mice and Simplified Human Intestinal Microbiota (SIHUMI) mice will be important to confirm the translational relevance of these findings. The durability of the antibody response and the establishment of long-term immunological memory were not assessed in the present study. If protection is primarily IgG-dependent, a resting period of at least 3–4 weeks should be incorporated to allow stabilization of the systemic humoral response. Future studies will therefore evaluate antibody persistence, memory B cell responses, the optimal immunization–challenge interval, and long-term protective efficacy to better define the feasibility of OppA-based vaccination strategies.

In conclusion, using a non-biased antibody library, we identified L2-1H11 as an OppA-reactive monoclonal antibody that cross-reacts with multiple *Salmonella* serotypes and recognized OppA as a candidate antigen associated with protective activity. Under our experimental conditions, L2-1H11 associated with the suppression of bacterial growth and respiratory activity, reduced neutrophil infiltration, and protection *in vivo*. These findings support the potential relevance of OppA as a candidate antigen against *Salmonella*, but further studies are required to determine whether OppA is the direct functional target mediating these effects and to define its native accessibility on intact bacteria. Furthermore, the non-biased antibody discovery strategy used here may facilitate the identification of protective antigens in other pathogens, accelerating the development of next-generation vaccines.

## Data Availability

The raw data supporting the conclusions of this article will be made available by the authors, without undue reservation.
