# Supplementary material for: Oligopeptide-binding protein A is a potential target for combating Salmonella infection
Source: Front Immunol. 2026 Oct 9;17:1943609. doi: 10.3389/fimmu.2026.1943609 (PMC13690942; doi:10.3389/fimmu.2026.1943609)
Supplement: Supplementary file 1 [file SupplementaryFile1.docx]

**Supplemental Information**

**Oligopeptide-binding protein A** **is a potential target for combating *Salmonella* infection**

Ken Yoshii, Koichiro Suzuki, Jun Adachi, Zhenyu Piao, Eri Node, Zilai Liu, Takahiro Nagatake, Koji Hosomi, and Jun Kunisawa

**Supplementary Fig. 1: Detection of *Salmonella*-specific IgG in the serum of mice immunized with heat-killed *Salmonella*,** *Related to Figure 1.*

Mice were immunized with heat-killed whole-cell preparations of *Salmonella* Enteritidis and *Salmonella* Typhimurium, and serum concentrations of serotype-specific antibodies were assessed by enzyme-linked immunosorbent assay. Data are presented as mean ± SD.

**Supplementary Fig. 2: Functional characterization of L2-1H11 antibody: enhanced phagocytosis, antibody-mediated protection, and effects on *Salmonella* Typhimurium virulence gene expression,** *Related to Figure 2*.

(A) A phagocytotic assay was performed using *Salmonella* Typhimurium conjugated with fluorescently labeled L2-1H11 antibody or an anti-*Salmonella* Typhimurium LPS antibody. Mean fluorescence intensity (MFI) is shown on the basis of flow cytometric analysis (n = 4). Data are combined from two independent experiments. Horizontal bars indicate mean value. ****P* < 0.001. (B) To determine whether the observed protection was antibody-mediated, L2-1H11 antibody (100 µg/mouse) was administered intravenously. The following day, mice were intravenously challenged with *Salmonella* Typhimurium (1.0 × 10⁶ CFU/mouse). Survival and body weight were monitored daily from the day of infection (n = 5). Data are representative of two independent experiments and are presented as mean ± SD. *P* < 0.05. (C–E) *Salmonella* Typhimurium cells were treated with 100 μg of isotype control or L2-1H11 antibody and incubated at 37°C for 1 h. Then, the expression of genes involved in *Salmonella* adhesion, invasion, and motility was determined by qRT-PCR (n = 4). Data are representative of two independent experiments and are presented as mean ± SD. One-way ANOVA (A), Two-tailed Student’s *t*-test (C-E), Log-rank (Mantel-Cox) test and (survival rate, B) and Repeated-measures two-way ANOVA (Body weight, B): N.S., not significant.

**Supplementary Fig. 3: Epitope mapping of *Salmonella*-derived OppA with L2-1H11 antibody,** *Related to Figure 3*.

An OppA peptide microarray was pre-stained with the secondary antibody in incubation buffer to investigate background interactions with the antigen-derived peptides that could interfere with the main assays. Incubation of further OppA peptide microarrays with the antibody sample at concentrations of 1 µg/ml, 10 µg/ml, and 100 µg/ml was followed by staining with the secondary antibody and read-out with an Innopsys InnoScan 710-IR Microarray Scanner at scanning gains of 50/10 (red/green). The additional HA peptides framing the peptide microarrays were subsequently stained with the control antibody as an internal quality control to confirm the assay performance and the peptide microarray integrity.

**Supplementary Fig. 4: Changes in amino acid levels in *Salmonella* cells after addition of L2-1H11 antibody,** *Related to Figure 4.*

(A) The indicated antibody (100 µg) was added to a *Salmonella* Typhimurium culture, and a time-course analysis of the intracellular amounts of major amino acids was conducted by liquid chromatography–tandem mass spectrometry (n = 4). (B, C) *Salmonella* Enteritidis was treated with 100 µg of either isotype control or L2-1H11 antibody. Oxygen consumption rate (OCR) and extracellular acidification rate (ECAR) were measured by flux analyzer (n = 4). (D) *Salmonella* Typhimurium cells were treated with 100 μg of isotype control, L2-1H11 antibody, or anti-*Salmonella* Typhimurium LPS antibody. After transfer to M9 minimal medium, oxygen consumption rate (OCR) was measured by flux analyzer (n = 4). Data are representative of two independent experiments and are presented as mean ± SD. Two-tailed Student’s *t*-test: **P* < 0.05.

**Supplementary Fig. 5:** **Changes in amino acid levels in** ***Escherichia coli* cells after addition of L2-1H11 antibody,** *Related to Figure 4***.**

The indicated antibody (100 µg) was added to an *E*. *coli* culture, and a time-course analysis of the intracellular amounts of major amino acids was conducted by liquid chromatography–tandem mass spectrometry (n = 4). Data are representative of two independent experiments and are presented as mean ± SD. Two-tailed Student’s *t*-test: N.S., not significant.

**Supplementary Fig. 6: Changes in protein synthesis levels in *Salmonella* cells after addition of L2-1H11 antibody,** *Related to Figure 4***.**

*Salmonella* Typhimurium was treated with the indicated antibody (100 μg) and incubated at 37°C for 1 h. Then, protein synthesis in the bacterial cells was analyzed by pulse–chase assay (n = 4). Data are representative of two independent experiments and are presented as mean ± SD. RFU, relative fluorescence units. Two-tailed Student’s *t*-test: N.S., not significant.

**Supplementary Fig. 7: Changes of energy metabolism in *Escherichia coli* cells after addition of L2-1H11 antibody,** *Related to Figure 4***.**

(A) The indicated antibody (100 µg) was added to an *E. coli* culture, and a time-course analysis made of the relative amounts of representative metabolites of glycolysis (lactate and pyruvate) and the TCA cycle (n = 4). (B, C) By using an XF24 extracellular flux analyzer, OCR and ECAR were measured in *E. coli* cells treated with isotype control or L2-1H11 antibody (100 µg) (n = 4). (D) ATP concentration in *E. coli* cells treated with isotype control or L2-1H11 antibody (100 µg) (n = 4). Data are representative of two independent experiments and are presented as mean ± SD. Two-tailed Student’s *t*-test: N.S., not significant.

**Supplementary Fig. 8: Subcutaneous immunization of mice with OppA did not suppress intestinal inflammation caused by *Salmonella* infection,** *Related to Figure 5.*

(A) Mice subcutaneously immunized with phosphate-buffered saline (mock) or OppA were orally infected with *Salmonella*, and the body weight was measured daily for 2 weeks post-infection (n = 10). (B) Number of *Salmonella* in feces. Fecal samples were collected on day 4 post-infection, suspended in LB broth, and the supernatant of the fecal slurry was plated onto *Salmonella-Shigella* agar, incubated overnight, and CFUs were counted. (C) Fecal samples were collected daily, and the concentration of myeloperoxidase (MPO), which is specifically secreted by neutrophils, was quantified by enzyme-linked immunosorbent assay (n = 4). Data are representative of two independent experiments and are presented as mean ± SD. Two-tailed Student’s *t*-test (B), repeated-measures two-way ANOVA (A, C): N.S., not significant.

**Supplementary Fig. 9: Oral immunization of mice with OppA induces *Salmonella*-specific IgG production and increases body weight after *Salmonella* infection,** *Related to Figure 6***.** (A) Detection of *Salmonella*-specific serum IgG antibody by enzyme-linked immunosorbent assay (n = 10) in mice orally immunized with phosphate-buffered saline (mock) or OppA three or four times in the following week. (B) Mice orally immunized with phosphate-buffered saline (mock) or OppA were orally infected with *Salmonella*, and body weight was measured daily for 2 weeks post-infection (n = 10). Data are representative of two independent experiments and are presented as mean ± SD. Two-tailed Student’s *t*-test (A) and repeated-measures two-way ANOVA (B): *****P* < 0.0001. N.S., not significant.

**Supplementary Table. 1: Proteomic analysis of molecules recognized by L2-1H11 antibody**

**Supplementary Table. 2: Surveillance data on non-typhoidal *Salmonella* reported by the National Institute of Infectious Diseases in the Infectious Agents Surveillance Report**
